# Digital health behaviour change interventions in severe mental illness: a systematic review

**DOI:** 10.1017/S0033291723002064

**Published:** 2023-11

**Authors:** Chelsea Sawyer, Grace McKeon, Lamiece Hassan, Henry Onyweaka, Luis Martinez Agulleiro, Daniel Guinart, John Torous, Joseph Firth

**Affiliations:** 1 Division of Psychology and Mental Health, The University of Manchester, Manchester Academic Health Science Centre, Manchester M13 9PL, UK; 2 School of Population Health, University of New South Wales, Randwick, NSW 2052, Australia; 3 Discipline of Psychiatry and Mental Health, University of New South Wales, Randwick, NSW 2052, Australia; 4 Department of Psychiatry, Harvard Medical School, Boston, MA, USA; 5 Department of Psychiatry, Massachusetts General/Mclean Hospital, Boston, MA, USA; 6 Department of Child and Adolescent Psychiatry, NYU Grossman School of Medicine, New York, NY, USA; 7 Hospital del Mar Research Institute, Institut de Salut Mental, Hospital del Mar, Barcelona, Spain; 8 Centro de Investigacion Biomedica en Red de Salud Mental (CIBERSAM), Spain; 9 Department of Psychiatry, the Donald and Barbara Zucker School of Medicine at Hofstra/Northwell, New York, USA; 10 Department of Psychiatry, Institute for Behavioral Science, Feinstein Institutes for Medical Research, Manhasset, NY, USA; 11 Zucker School of Medicine at Northwell/Hofstra, New York, NY, USA; 12 Department of Psychiatry, Beth Israel Deaconess Hospital, Boston, MA, USA; 13 Greater Manchester Mental Health NHS Foundation Trust, Manchester Academic Health Science Centre, Manchester M13 9PL, UK

**Keywords:** digital health, schizophrenia, smoking, exercise, diet, sleep

## Abstract

The use of digital technologies as a method of delivering health behaviour change (HBC) interventions is rapidly increasing across the general population. However, the role in severe mental illness (SMI) remains overlooked. In this study, we aimed to systematically identify and evaluate all of the existing evidence around digital HBC interventions in people with an SMI. A systematic search of online electronic databases was conducted. Data on adherence, feasibility, and outcomes of studies on digital HBC interventions in SMI were extracted. Our combined search identified 2196 titles and abstracts, of which 1934 remained after removing duplicates. Full-text screening was performed for 107 articles, leaving 36 studies to be included. From these, 14 focused on physical activity and/or cardio-metabolic health, 19 focused on smoking cessation, and three concerned other health behaviours. The outcomes measured varied considerably across studies. Although over 90% of studies measuring behavioural changes reported positive changes in behaviour/attitudes, there were too few studies collecting data on mental health to determine effects on psychiatric outcomes. Digital HBC interventions are acceptable to people with an SMI, and could present a promising option for addressing behavioural health in these populations. Feedback indicated that additional human support may be useful for promoting adherence/engagement, and the content of such interventions may benefit from more tailoring to specific needs. While the literature does not yet allow for conclusions regarding efficacy for mental health, the available evidence to date does support their potential to change behaviour across various domains.

## Introduction

Along with poor mental health, people with severe mental illness (SMI), such as bipolar disorder, schizophrenia, and other psychotic disorders, show elevated risks of engaging in adverse health behaviours (Carney, Cotter, Bradshaw, Firth, & Yung, 2016; Firth et al., 2019). For example, in comparison with the general population people with SMI are more likely to smoke cigarettes (Prochaska, Das, & Young-Wolff, 2017), are less physically active, and have higher daily calorie and sodium intake (Teasdale et al., 2019; Vancampfort et al., 2017). This may be partly attributable to the psychotropic medications used to treat SMI, as antipsychotics have been found to increase appetite, delay satiety signalling, and cause sedation (Mazereel, Detraux, Vancampfort, Van Winkel, & De Hert, 2020). Finding novel ways to promote healthy lifestyles in SMI is crucial for reducing morbidity and mortality (Firth et al., 2019), with increasing evidence to suggest this could also improve mental health outcomes (Firth et al., 2020; Pape, Adriaanse, Kol, van Straten, & van Meijel, 2022).

Health behaviour change (HBC) interventions include a broad range of psychological techniques, and target modifiable health behaviours such as diet, physical activity, smoking, sleep, substance or alcohol use, and medication adherence. Traditional face-to-face HBC, while ideal in many respects, interventions are resource intensive (Bennett & Glasgow, 2009) and can be impacted by the capability and capacity of the person delivering the intervention. Interest in online HBC (web-based and smartphone) has grown rapidly in popularity (Arigo et al., 2019), given their potential to improve access to HBC for people with SMI, without relying on costly face-to-face interventions (Young et al., 2017). Previously, there have been concerns that people with SMI may experience socio-economic barriers – such as unstable housing, low income, and unemployment – which may limit their access to the internet and online interventions (Borzekowski et al., 2009). Encouragingly however, smartphone and internet use is increasing among those with SMI (Firth et al., 2016; Thomas, Foley, Lindblom, & Lee, 2017; Trefflich, Kalckreuth, Mergl, & Rummel-Kluge, 2015).

While previous reviews have focused on the feasibility and acceptability of digital interventions generally for symptom management and relapse prevention in SMI (Naslund, Marsch, McHugo, & Bartels, 2015b), there is still limited understanding of how digital HBC could work to improve outcomes specifically in this population. Therefore, this review aimed to systematically identify and evaluate the current evidence around the feasibility, acceptability, and effectiveness of digital HBC for not only physical health, but also broader behavioural and psychological well-being outcomes, in people with SMI.

Specifically, this review addressed the following research questions: (i) are digital approaches towards delivering HBC feasible and acceptable for people with SMI?; (ii) can digital HBC for people with SMI change health-related behaviour?; (iii) can digital HBC for people with SMI improve health outcomes?; and (iv) what specific intervention components and strategies influence user engagement with digital HBC interventions in people with SMI?

## Methods

The Preferred Reporting Items for Systematic Reviews and Meta-Analyses (PRISMA) checklist for reporting systematic reviews (Moher, Liberati, Tetzlaff, Altman, & PRISMA Group, 2009) guided this review, which was pre-registered on the online review protocol database, PROSPERO (CRD42021261267).

### Search strategy

A systematic literature search was conducted in January 2022 using the following databases: Cochrane Central Register of Controlled Trials; Health Technology Assessment; AMED (Allied and Complementary Medicine); APA PsycInfo; Embase; and MEDLINE^®^, using the following keyword search algorithm: [psychosis OR psychotic OR schizophr* OR severe mental OR serious mental OR bipolar] AND [Behaviour change OR Behavior change OR behavioural change OR behavioral change OR Lifestyle OR Healthy Living OR Health Behaviour OR Health Behavior OR physical activity OR exercise OR smok* OR tobacco OR sexual health OR Sleep OR Alcoho* OR diet* OR Sedentary OR substance abuse OR weightloss OR weight loss OR obes*] AND [online or web-based or app-based or Internet or e-Health or mhealth or smartphone or mobile phone or iphone or android or wearable or digital].

Searches were restricted to publication in English language in peer-reviewed journals and all articles were included regardless of publication date. Reference and citation list searches were also conducted to search for additional studies, alongside a basic search of Google Scholar and the *Journal of Medical Internet Research* (JMIR).

### Eligibility criteria

English language articles were included. Randomised controlled trials (RCTs), non-RCTs, pilot studies, feasibility studies, quasi-experimental studies, and qualitative studies examining the feasibility, acceptability, or effectiveness and other outcomes of a digital HBC, delivered online via computer smartphone apps, social media and/or ‘wearable’ formats, among people with SMI were eligible.

For the purpose of this review, ‘SMI populations’ included any groups of individuals (of any age) diagnosed and/or receiving treatment for bipolar or psychotic disorders. Studies of non-entirely SMI samples were included, if either (i) where data pertaining to the SMI sub-sample were reported separately, or, (ii) where the overall sample contained over two-thirds of individuals diagnosed/treated for SMI.

Studies that reported changes in health behaviours relevant to physical health and overall well-being (such as smoking, substance use, sleeping, diet, physical activity, and sexual behaviours) as primary or secondary outcomes were included. Studies will be eligible for inclusion if they deliver a behavioural change intervention fully online, or where the digital technology forms a well-defined and central part of a multi-component intervention. Interventions in which the HBC aspect only made up a tangential or minor part of the intervention, or where relevant technological aspects were limited to text messages, emails or phone calls, were excluded.

### Study selection process

Initial screening of titles and abstracts was conducted by one reviewer (C.S.). Two reviewers (C.S. and G.M.), who were blind to each other's review, independently reviewed all full-text articles meeting the inclusion criteria (interrater agreement: 83%). A third reviewer (J.F.) resolved any disagreements between the two reviewers.

### Data extraction

Data were independently extracted by two reviewers (C.S. and G.M.), using a pre-determined data extraction form specifically designed for this review. The extraction form collected the following data: (i) study information (sample size, mean age of participants, diagnostic information, and study design); (ii) intervention features (intervention platform, app/programme name, trial/feasibility details, regularity of instructed use, digital intervention summary, any additional intervention components, and details of the control condition); and (iii) effects on behaviour and health outcomes (changes in behaviour, changes in physical and/or mental health before and after interventions).

Given there are no established standards for assessing feasibility, acceptability, and usability (Greenhalgh et al., 2017; Jacob, Sezgin, Sanchez-Vazquez, & Ivory, 2022), measures were chosen from validated scales [e.g. the System Usability Scale (SUS) (Hyzy et al., 2022)] and in line with prior research (Balaskas, Doherty, Schueller, & Cox, 2021). Feasibility measures included recruitment rates, attrition to study, reasons for refusal or ineligibility, and adherence to intervention. Acceptability measures included usage data (e.g. duration of use, modules completed, etc.) and user perspectives from interviews and quantitative questionnaires (e.g. regarding relevance of content and readability). Usability measures included the SUS, task scores, and interview comments regarding design, layout, and/or other aspects of the user interface. Finally, behavioural outcomes included intervention effect sizes and/or changes in target behaviour, depression, and/or anxiety.

### Data synthesis

Due to variations in design, intervention approaches, and primary outcome measures it was not appropriate to conduct a meta-analysis and therefore quantitative findings were synthesised narratively (Liberati et al., 2009). For qualitative studies, themes were identified and the detailed analytical narratives reported within the text were summarised, following the principles of thematic synthesis (Thomas & Harden, 2008). Mixed methods studies contributed separately to both types of synthesis. The data in each section were different and were therefore not double counted.

## Results

**Figure 1.**
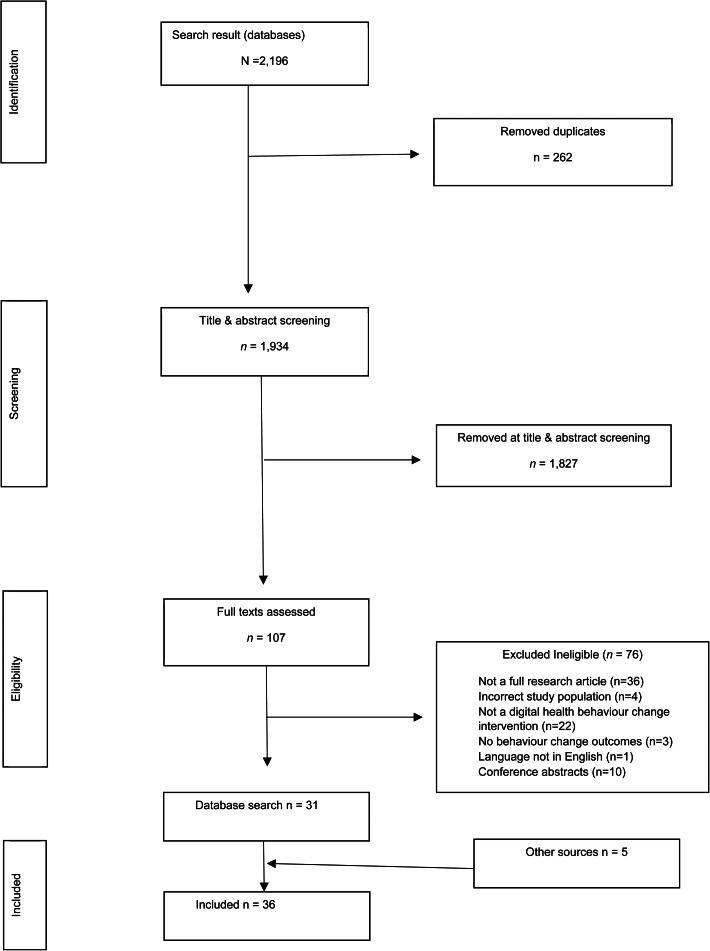
PRISMA flowchart of study selection.

Overall, 2196 results were identified from the databases. Figure 1 presents a PRISMA flowchart of study selection procedures. After removing duplicates (*N* = 262), 1934 titles and abstracts were screened. Following initial screening, 1827 articles were removed, leaving 107 full texts to be reviewed. A further 76 were then excluded, leaving 31 papers. Five additional articles were found through separate literature searches of reference lists and Google Scholar, yielding 36 papers in total.

### Study characteristics

Tables 1–3 summarise the characteristics of selected studies, including descriptions of participants and interventions. There were 14 pilot RCTs, 14 non-RCTs, three qualitative studies, and five mixed-method studies. The majority (*N* = 35) of studies were conducted in the United States, with only one study conducted in the UK, Australia, Portugal, Netherlands, and Canada. Fourteen of the studies consisted of mainly White participants. Sample sizes ranged from 5 to 2570 participants. Schizophrenia was the most common diagnosis in smoking and physical activity studies, whereas bipolar disorder was the most common diagnosis for ‘other behaviours’ (Tables 1–3).

**Table 1.** Descriptive characteristics for the studies on digital interventions in SMI for smoking

Authors, year Country	Study design study duration	Target sample, *N*	Diagnosis (%)	Mean age (s.d.; range) Gender (male %) Ethnicity	Intervention mode	Number phases Intervention duration	Control condition	Details about intervention
Browne et al. (2021) United States	Pilot RCT, 16 weeks	⩾18 years old with ICD-10 diagnosis of an SMI and smoke ⩾5 cigarettes daily. *N* = 62 LTQ *n* = 33 QG *n* = 29	SZ 24% BP 48% MDD 27%	LTQ 46.1 (s.d. 11.3) years old QuitGuide 45.6 (s.d. 10.9) years old **Male:** LTQ 36% QuitGuide 45% **Ethnicity:** LTQ: 49% White; 42% African American; 3% Asian; 6% Multiracial; QuitGuide: 55% White; 38% African American; 3.5% Asian; 3.5% Multiracial	Digital [compared two apps; Learn to Quit (LTQ) *v.* QuitGuide]	Phase: n/a Duration: NR	None	LTQ: 28 modules that provide knowledge, skills, and recommendations for smoking cessation designed for persons with SMI. NCI QuitGuide: includes health information about smoking, tracking tools (e.g. for cravings), and advice for quitting. All had 8 weeks NRT All received a smartphone and data.
Brunette et al. (2011) United States	Quasi-experimental design 2 months	Adult smokers with an SMI who are receiving care in supported housing or psychiatric rehabilitation. *N* = 41 I *n* = 21 C *n* = 20	SZ 34.1%	I: 47 (s.d. = 9) years old C: 48 (s.d. = 11) years old **Male:** I 67%; C 65% **Ethnicity:** African American I 95%; C 83%	Digital (website electronic decision support system)	Phase: n/a Duration: use once within 2 weeks.	Waitlist	Electronic decision support system (30–90 min): videorecorder narrator, who identifies as a former smoker with SMI, guides users through the programme and encourages them to quit smoking. The users answer questions about their smoking behaviour. The user blows into a carbon monoxide meter, which measure severity of nicotine dependence. Feedback is provided about the users’ health risks. Users answer questions about their personal views of smoking. A summary in the form of a decision balance is created.
Brunette et al. (2012) United States	Usability and qualitative study 1 day	Adult smokers receiving treatment for an SMI. *N* = 16	SZ 75% BP 6.3% MDD 6.3% Other 12.5%	49.9 (8.9) years old **Male:** 62.5% **Ethnicity:** 93.8% African American	Digital (four websites) becomeannex.org, pmusa.com, Smokefree.gov, then whyquit.com	Phase: n/a Duration: 1 day	None	n/a assessed whether four smoking cessation websites met usability guidelines
Brunette et al. (2016) United States	Usability study 8 weeks	Adult smokers with psychotic illnesses who are interested in quitting in the next month. *N* = 11 Group 1 *n* = 5 Group 2 *n* = 6	SZ spectrum 91%	49 (10) years old **Male**: 45% **Ethnicity:** African American 33%	Digital (website prototype Let's Talk About Quitting Smoking)	Phase: 2 Phase 1: prototype website used in office Phase 2: prototype website at home Duration: 4 weeks each phase	None	Website was adapted for people with SMI. Website consisted of: eight interactive modules online forum support group a ‘library’ of educational materials tailored to SMI
Brunette et al. (2018) United States	Pilot RCT 3 months	Daily smokers aged 18–30 with schizophrenia. All *N* = 81; LTAS *n* = 30 NCI *n* = 28 Control *n* = 23	SZ spectrum 43.2% Severe mood and anxiety disorders 57.8%	All 24.2 (3.6) years old LTAS 23.5 (3.9) years old NCI 25.0 (3.2) years old Control 26.1 (3.3) years old **Males:** 77.8% **Ethnicity:** 65.4% White; 25.9% Black; 8.6% Other	Digital (website either LTAS or NCI patient education handout presented on a laptop)	Phase: n/a Duration: delivered on a laptop computer in a single session.	None	LTAS: tailored for those with an SMI. Focuses on financial and social impacts of smoking and quitting. NCI: provides information about smoking-related diseases and smoking cessation treatments.
Brunette et al. (2019) United States	Pilot 8 weeks	⩾17 years old daily smokers, who had a diagnosis of SMI and was an outpatient who want to quit in the next month. *N* = 20	SZ spectrum 60%	46 (13.6) years old **Male**: 70% **Ethnicity:** 80% White	Digital (website LTAS)	Phase: n/a Duration: 8 weeks	None	Practised accessing website, once able to use they were provided with a laptop and jetpack for wireless internet and asked to use website 5× a week. 12 modules, with 48 interactive sessions (intended for daily use) Library of easy to read information 2 week supply of NRT
Brunette et al. (2020) United States	RCT 6 months	Adult daily smokers with schizophrenia spectrum disorders. Smokers were excluded if they had recently (past month) used evidence-based smoking cessation treatment. *N* = 162 LTAS *n* = 78 NCI *n* = 77	SZ spectrum 100%	45.91 (11.3) years old **Male:** 66.7% **Ethnicity:** 29% White; 53.1% Black; 17.9% mixed/other	Digital (website either LTAS or NCI education handout presented on a laptop)	Phase: n/a Duration: delivered on a laptop computer in a single session.	None	LTAS: tailored for those with an SMI. Focuses on financial and social impacts of smoking and quitting. 3 modules assessment/feedback, quitting intentions, and education about smoking cessation treatments NCI: provides information about smoking-related diseases and smoking cessation treatments.
Ferron et al. (2011) United States	Qualitative study Not stated	Daily smokers with mental health condition. Phase 1 *N* = 41 Phase 2 *N* = 8 Phase 3 *N* = 22	SMI 100%	Age: NR **Male:** 63% **Ethnicity:** 69% White; 31% African American	Digital (website does not state name)	Phases: 3 Duration: 1 session per phase	None	Not included.
Gowarty et al. (2021a) United States	Usability study 2 weeks	18–35 receiving treatment for SMI and a smartphone user. *N* = 17	Psychotic disorder 41%	29 (4) years old **Male:** 59% **Ethnicity:** 94% White	Digital (app assigned to either QuitGuide or quitSTART app)	Phase: n/a Duration: 2 weeks of independent use	None	Both apps have many similar features (such as set notifications based on time or location), but their design and content differ. QuitGuide utilises: a linear layout, darker colours, text heavy with minimal graphics. Additional feature journal entries and users can read a text-only guide on how to quit smoking. quitSTART utilises: a more complex layout, brighter colours, informal language, large symbols, and few words within the icons, information presented on swipe able cards, 7 distraction games, check-in notifications are automated to ask users ‘how many cigarettes they have smoked since the last check-in’.
Gowarty et al. (2021b) United States	Usability study 2 weeks	18–35 receiving treatment for SMI and a smartphone user. *N* = 17	Psychotic disorder 41%	29 (4) years old **Male:** 59% **Ethnicity:** 94% White	Digital (compared two apps, QuitGuide *v.* quitSTART)	Phase: n/a 2 weeks independent use	None	Both apps have many similar features (such as set notifications based on time or location), but their design and content differ. QuitGuide utilises : a linear layout, darker colours, text heavy with minimal graphics. Additional feature journal entries and users can read a text-only guide on how to quit smoking. quitSTART utilises: a more complex layout, brighter colours, informal language, large symbols, and few words within the icons, information presented on swipe able cards, 7 distraction games. Check-in notifications are automated to ask users ‘how many cigarettes they have smoked since the last check-in’.
Heffner et al. (2018) United States	RCT 12 months	Daily smokers with a mental health condition. *N* = 2570	BP 8.6% SZ-AFF 30.5%	46.2 (13.3) years old **Male:** 20% **Ethnicity:** 73% White; 8% Hispanic	Digital (website compared WebQuit Plus *v.* Smokefree.gov)	Phase: n/a Duration: 12 months	None	WebQuit Plus: the website is ACT based and has four parts that help users: (1) make a quit plan, (2) develop awareness of smoking triggers, (3) develop acceptance-based coping skills to handle triggers, and (4) identify and engage personal values and self-compassion to support long-term abstinence. Smokefree.gov is a multimedia website that includes (1) guidance on setting a quit date, preparing to quit, identifying and coping with triggers, and staying motivated; (2) interactive content, such as screening questionnaires for depression and nicotine dependence; and (3) information about the health effects of smoking, presented in text and graphic form. All received up to 4 texts a day, for the first 28 days. Text message content differed between arms.
Heffner et al. (2020) United States	Pilot RCT 1 month	Bipolar I or II disorder. All *N* = 51 Smokefree.gov *n* = 26 ACT-based WebQuit *n* = 25	BP 88%	49.0 years old (10.8) **Male:** 55%; **Ethnicity:** 73% White; 8% Hispanic	Digital (website compared WebQuit Plus *v.* Smokefree.gov)	Phases: n/a Duration: 1 month	None	WebQuit Plus: the website is ACT based and has four parts that help users: (1) make a quit plan, (2) develop awareness of smoking triggers, (3) develop acceptance-based coping skills to handle triggers, and (4) identify and engage personal values and self-compassion to support long-term abstinence. Smokefree.gov is a multimedia website that includes (1) guidance on setting a quit date, preparing to quit, identifying and coping with triggers, and staying motivated; (2) interactive content, such as screening questionnaires for depression and nicotine dependence; and (3) information about the health effects of smoking, presented in text and graphic form. Both interventions consisted of (1) weekly email reminders, (2) daily text messages for 70 days, and (3) nicotine patch for 8 weeks.
Klein et al. (2019) Australia	Qualitative study Duration: Two consecutive interviews conducted (1 + 1.5 h)	Adults with SMI who had attempted to quit smoking in the last 12 months or ex-smokers. *N* = 12	SZ 75% BP 8% Borderline personality disorder 17%	Median: 47.5 (range 31–53) years old **Male:** 67% **Ethnicity:** NR	App (Kick.it)	Stages: 2 Duration: NR	None	Four core features are: smoke and crave profile – tracks user's real-time smoking and quitting behaviours in real and delivers quit strategies, Kick.it stack contains education and strategies to quit, social network contains chatrooms, community feed, mission and treatment goal.
Medenblik et al. (2020) United States	Pilot RCT 6 months	Adults aged between 18 and 70 years old, who smoke at least 10 cigarettes per day and have smoked for ⩾1 year. *N* = 35	SZ 44% SZ-AFF 53% PNOS 3%	48.2 (9.9) years old **Male:** 80% **Ethnicity:** 12% White; 62% Black/African American; 24% multiple races; 2% American Indian/Alaskan native	Blended: in-person and phone counselling and apps (mcm readings uploaded on an app)	Phase: n/a Duration: NR	Intensive treatment comparison, which consisted of: 5 CBT counselling sessions and pharmacotherapy.	iCOMMIT consists of: (i) smartphone-based application (mCM) to upload video recordings confirming abstinence and receive compensation. (ii) CBT counselling: 5 sessions and a workbook, provided per cohort. (iii) pharmacotherapy: prescribed bupropion and started prescription 2 weeks before quit date. Daily for days 1–7 and twice a day for 6 months, following quit date.
Vilardaga et al. (2016) United States	Usability study Duration: not stated	Adult with SMI. *N* = 5	SZ 40% BP 60%	51.2 (4.27) years old **Male:** 100% **Ethnicity:** 20% White	Digital (QuitPal app)	Phase: not stated Duration: 3 days Day 1 introduced to QuitPal Days 2–3 field test the app Then invited for an interview		QuitPal
Vilardaga et al. (2018) United States	Mixed methods Duration: not stated	Daily smokers recruited from outpatient MH clinic. *N* = 5	SMI 100%	44 (7.5) years old **Male:** 42.9% **Ethnicity:** 60% White; 20% African American; 20% mixed	Digital (apps LTQ *v.* QuitGuide)	Phases: 3 AB crossover intervention, B-phase training designs and bi-phasic, AB single-case design Phase duration: ranged from 7 to 23 days	None	LTQ: developed for those with an SMI. The app has 28 modules (14 lessons and 14 skills) that provide knowledge, skills, and recommendations for smoking cessation. QuitGuide: developed for general population. The app includes health information about smoking, tracking tools (e.g. for cravings), and advice for quitting.
Vilardaga et al. (2019) United States	Case studies with crossover AB interventions Duration: not stated	Adults with SMI. *N* = 7	SZ 14.2% PNOS 28.6% Mood disorder 57.2%	45 (9.5) years old **Male:** LTQ 36%; QuitGuide 45% **Ethnicity:** 71.4% White; 28.6% mixed	Digital (compared two apps LTQ *v.* QuitGuide)	Phases: total 7 5 which were user-centred, design; preliminary testing, efficacy, effectiveness Last 2 phases not user-centred. Phase duration	None	LTQ: developed for those with an SMI. The app has 28 modules (14 lessons and 14 skills) that provide knowledge, skills, and recommendations for smoking cessation. QuitGuide: developed for general population. The app includes health information about smoking, tracking tools (e.g. for cravings), and advice for quitting.
Vilardaga et al. (2020) United States	Pilot RCT 16 weeks	Adults with SMI and stable housing, who smoke >5 cigarettes a day. *N* = 62 LTQ *n* = 33 QG *n* = 29	RMD 27% BP 48% SZ spectrum 24%	LTQ 46.1 years old (11.3) QuitGuide 45.6 years old (10.9) **Male:** 40% **Ethnicity:** LTQ: 100% not Hispanic/Latin QuitGuide: 97% not Hispanic/Latin	Digital (LTQ app *v.* QuitGuide app)	Phase: n/a 16 weeks	None	LTQ: developed for those with an SMI. The app has 28 modules (14 lessons and 14 skills) that provide knowledge, skills, and recommendations for smoking cessation. (i) each day participants could complete 1 skill and 1 lesson module, (ii) smartphone and data provided, (iii) 8-week course of transdermal nicotine patches. QuitGuide: developed for general population. The app includes health information about smoking, tracking tools (e.g. for cravings), and advice for quitting. (i) app, (ii) smartphone and data provided, (iii) 8-week course of transdermal nicotine patches.
Wilson et al. (2019) United States	Successive cohort design Duration: not stated	Aged 18–70, smoke ⩾10 cigarettes daily and smoking for ⩾1 year, and met criteria for schizophrenia, schizoaffective disorder, or another psychotic disorder. Cohort 1 *N* = 5 Cohort 2 *N* = 8	SZ 46.7% SZ-AFF 33.3% PNOS 6.7%	47.8 years old (11.0; 26–63) **Male:** 53.3% **Ethnicity:** 61.5% African American; 7.7% White; 15.4% mixed	Blended: digital (Stay quit coach app and mcm readings uploaded on an app) and in-person and telephone counselling	Phase: two cohorts Duration: 3 months each cohort	None	iCOMMIT consists of: (i) stay Quit Coach app, (ii) smartphone-based application (mCM) to upload video recordings confirming abstinence and receive compensation, (ii) CBT counselling: 5 sessions and a workbook, provided per cohort, (iv) pharmacotherapy: prescribed bupropion and started prescription 2 weeks before quit date. Daily for days 1–7 and twice a day for 6 months, following quit date.

BD, bipolar disorder; CBT, cognitive behavioural therapy; FTDN, Fagerström test for nicotine dependence; LTAS, Lets Talk About Smoking; LTQ, Learn To Quit; MDD, major depressive disorder; MH, mental health; NA, not applicable; NCI, National Cancer Institute; NR, not reported; NRT, nicotine replacement therapy; NS, not significant; PNTS, prefer not to say; PNOS, psychosis not otherwise specified; PPA, point prevalence abstinence; QG, quit guide; RCT, randomised controlled trial; RMD, recurrent major depression; s.d., standard deviation; SMI, serious mental illness; SZ, schizophrenia; SZ-AFF, schizoaffective disorder; SUS, System Usability Scale; TAU, treatment-as-usual; mcm, smartphone-based application contingency management, iCOMMIT, multi-component mobile-enhanced treatment for smoking cessation.

**Table 2.** Descriptive characteristics for the studies on digital interventions in SMI for physical activity

Authors, year Country	Study design Study duration	Target sample, *N*	Diagnosis	Mean age (s.d.; range) Gender (male %) Ethnicity	Intervention mode	Intervention duration number phases	Control condition	Intervention components
Aschbrenner et al. (2015) United States	Mixed-methods repeated measure design 6 months	Adults with an SMI diagnosis and a BMI ⩾25 *N* = 10	SZ 20% SZA 10% BP 10% MDD 60%	46.6 years old (8.7; 30–57) Male: 10% Ethnicity: 90% White	Blended – digital elements: wearable and app	24 weeks in 3 phases: phase 1 (weeks 1–4): training on tracking PA and smartphone use. Phase 2 (weeks 5–12): peer health coach engagement, focusing on PA and healthy eating. Phase 3 (weeks 13–24): peer health coach reviews behaviours and outcomes.	None	**In-person:** (i) fitness club membership (ii) 8–10 personalised fitness training sessions (iii) one-on-one and group-based peer health coaching sessions **Digital:** (iv) weekly motivational text messages (v) wearable Wearable and smartphone was provided
Aschbrenner et al. (2016a) United States	Pilot RCT 6 months	Adults with SMI and BMI ⩾30 *N* = 32	SZ 22% MDD 44% BP 34%	48.8 years old (11.9) Male: 44% Ethnicity: 97% White	Blended – digital elements: wearable, app, and social media	24 weeks n/a	None	**In-person:** (i) 1×/week 1-h group weight management sessions facilitated by two lifestyle coaches (ii) 2×/week (optional) 1-h group exercise sessions led by a certified fitness trainer **Digital:** (iii) mHealth technology – receive text message reminders about programme activities (iv) social media (Facebook group) (v) wearable (Fitbit Zip) Wearable and phone was provided
Aschbrenner et al. (2016b) United States	Feasibility study 6 months	⩽21 years old with an SMI and BMI ⩾30 *N* = 13	SZ 27% BD 46% MDD 27%	48.8 years old (10.6; 21–58) Male: 27% Ethnicity: 91% White	Blended – digital elements: text reminders, wearable, app, and social media	24 weeks	None	**In-person:** (i) weekly 90-min weight management group sessions* (ii) 2×/week (optional) 1-h group exercise sessions **Digital:** (iii) social media* – weekly lessons from weight management sessions were uploaded onto to the Facebook group and had group discussion (iv) wearable (Fitbit zip) and Fitbit app (v) text reminders regarding sessions and to increase activity Wearable and IPhone was provided *Wellness couches (individuals with SMI who previously participated in PA intervention) were involved
Aschbrenner et al. (2021) United States	RCT 12 months	18–35 years old with an SMI and BMI ⩾25 *N* = 150	SZ or SZA 40% BD 20% Psychotic disorder not otherwise specified 4% Depression 12% PTSD 17% Anxiety 3% Mood disorder not specified 4%	28.4 (4.5) years old Male: 45% Ethnicity: 55% White 26% Black 1% Asian 8% >1 30% Hispanic	**PeerFIT:** Blended – digital elements: wearable, app, and social media BEAT – digital: wearable and app (Fitbit) and smart body weight scales	**PeerFIT:** 12 months 2× 6 month phases 1st (intensive) phase consisted of twice-weekly group (3–12 members) meetings 2nd (maintenance) phase consisted of weekly exercise sessions **BEAT:** 12 months One phase	None	**PeerFIT** **In-person:** (i) group meetings (1st phase) and exercise sessions (2nd phase) **Digital:** (ii) wearable and app (Fitbit Z) (iii) social media (private Facebook group) (iv) weekly text messages for reminders, encouragement, and boost motivation (3–5 texts per week) from coaches **BEAT remote:** (i) one-on-one mHealth coaching delivered by health coaches via telephone calls (5 per monthly 30-min telephone coaching calls) (ii) text messaging (iii) wearable activity trackers (Fitbit) (iv) smart body weight scale was provided (v) weekly text messages (3–5 texts per week) from coaches, motivational, and persuasive to change behaviour in 6 lifestyle areas
Campos et al. (2015) Portugal	Feasibility study and quasi-experimental trial 8 weeks	Adults diagnosed with schizophrenia *N* = 32 (16 in each)	SZ: 100%	I: 39.77 years old (9.2) C: 39 years old (5.60) Male: I: 76.92% C: 68.75% Ethnicity: NR	Digital: Exergame (Microsoft Kinect online)	8 weeks	TAU	**Exergame, Microsoft Kinect:** (i) 2×/week 20-min game sessions **at outpatient centre** Exergame had 20 levels in each of 3 games, which involved whole body movements to control a virtual avatar (e.g. dodging obstacles and stomping)
Looijmans et al. (2019) Netherlands	RCT 12 months	SMI patients with at least one metabolic risk factor All *N* = 244 I *n* = 140 C *n* = 104	Psychotic disorder 57.6% Personality disorder 26.3% Mood disorder 28.0% Anxiety disorder: 13.6%	46.1 (10.8) years old Male: 49.2% Ethnicity: NR	Blended – digital element (webtool)	12 months, two phases 1st phase (screening phase, consisting of 3 visits) 2nd phase (follow-up consisting of 23 visits which were conducted at regular care meetings)	TAU	26 fortnightly meetings between MH nurse and service user, using the web tool. 1st phase: patients and nurses used the web tool to make SMART goals, intervention lasted 6 months 2nd phase: patients progress towards their goal was reviewed and feedback provided and goals were revised
Macias et al. (2015) United States	RCT 4 weeks	Individuals with an SMI diagnosis and own a smartphones *N* = 10	SZ 40% BP 30% MDD 30%	Range 22–61 years old Male: 50% Ethnicity: 100% White	Digital (WellWave app)	4 weeks	None	**Digital**: app which prompts, records, and tracks walking (the date and time of each walk, duration in minutes, step count, and speed)
Muralidharan et al. (2018) United States	RCT 6 months	Veterans with SMI and (BMI) >30 or BMI >28 with weight gain of ⩾10 lbs in the last 3 months *N* = 276	100% SMI	WebMOVE: 54.7 (8.9) years old MOVE-SMI: 53.7 (9.6) years old TAU: 54.2 (9.8) years old **Male:** WebMOVE 91% MOVE-SMI 92.6% TAU 97.7% **Ethnicity: WebMOVE:** 40% White 47% African American 1% Asian **MOVE-SMI:** 42% White 50% African American 4% Asian **TAU:** 39% White 53% African American 2% Asian	Digital (website; WebMOVE) *v.* in-person (MOVE-SMI)	6 months	TAU	**MOVE-SMI**: (i) 8 in-person individual sessions (involved goal setting, reviewing behaviour) (ii) 16 in-person group sessions (involved weekly weigh-ins, information, and review of progress/challenges) **WebMOVE** (a computerised version of MOVE-SMI with the same curriculum): (i) delivered via 30 online interactive modules (15 on diet; 15 on physical activity) (ii) received a pedometer (iii) peer coaches (individuals with an SMI) conducted weekly 25–30 min coaching calls (iv) access to online system and the peer coaching support
Naslund et al. (2015a, 2015b) United States	Feasibility study Duration: NR	Adults with an SMI and who are overweight/obese *N* = 10	SZ 30% BP 10% MDD 60%	47.7 (9.0; 30–58) years old Male: 10% Ethnicity: 90% White	Blended – digital elements (wearable and tracking app)	Duration: wore wearable for 80–133 days depending on date of enrolment	None	**In-person:** (i) weekly peer-led group and individual exercise and nutrition education sessions (ii) individual meetings with a certified fitness trainer **Digital:** (iii) wearable to sync with smartphone (iv) app to track behaviour Wearable (50% Fitbit Zip and 50% Nike FuelBand) and iPhone were provided
Naslund et al. (2016) United States	Feasibility study 6 months	Adults with an SMI and have a BMI ⩾30 *N* = 11	SZ 27% MDD 46% BP 27%	48.2 (11.2) years old Male: 27% Ethnicity: 100% White	Blended – digital elements (wearable, tracking app, and social media)	6 months	None	**In-person** at a community mental health centre: (i) 1×/week 90-min group weight management sessions focused on goal setting, self-monitoring, and problem solving delivered by lifestyle coach and wellness peers (ii) 2×/week optional 1-h exercise sessions led by fitness trainer **Digital:** (iii) mHealth technologies (regular text messages sent) (iv) a private Facebook group was introduced after 10 weeks (v) wearable A Fitbit and iPhone were provided
Naslund et al. (2018) United States	Feasibility study 6 months	⩾21 years old with an SMI and a BMI ⩾30 *N* = 25	SZ 20% BP 36% MDD 44%	49.2 (11.8) years old Male: 48% Ethnicity: 96% White	Blended – digital elements (wearable, tracking app, and social media)	6 months	None	**In-person** delivered at community mental health centre: (i) 1×/week group weight management session facilitated by two lifestyle coaches (ii) 2×/week optional group exercise sessions led by a fitness instructor **Digital:** (iii) received 3–5 text reminder messages (iv) Fitbit wearable (v) private Facebook group moderated by lifestyle and peer coaches A Fitbit was provided
Olmos-Ochoa et al. (2019) United States	Mixed method (RCT + Qual) 6 months	Veterans with SMI and BMI >30 or BMI >28 with weight gain of ⩾10 lbs in the last 3 months All *N* = 277 WebMOVE *n* = 93; MOVE-SMI *n* = 95; C *n* = 89 Interview: random subsample *N* = 48 (25.5%; 24 from each intervention)	100% SMI	WebMOVE: 54.7 (8.9) years old MOVE-SMI: 53.7 (9.6) years old C: 54.2 (9.9) years old **Male**: WebMOVE: 91.2%; MOVE-SMI: 92.6% C: 97.8% **Ethnicity:** **WebMOVE** 38% White 46% Black/African-American **MOVE-SMI** 38% White 45% Black/African-American **TAU** 36% White 51.7% Black/African-American	**WeBMOVE:** digital (website, calls, and pedometer) **MOVE-SMI**: in-person	Interviewed after 6 months on intervention	TAU	**WebMOVE – Digital:** (i) internet browser-based provision of 30 interactive educational modules (15 on diet; 15 on physical activity) (ii) pedometer (provided) (iii) monitoring weight and individualised virtual homework (iv) weekly telephonic peer coaching **MOVE-SMI** – **In-person:** (v) 24 60-min sessions (8 individual and 16 group) (vi) utilises handouts, motivational techniques, visual learning aids, behavioural rehearsal, repetition, goal setting, homework, and diet plans
Sylvia et al. (2021) United States	Comparative effectiveness study 16 weeks	18–65 years old with a self-reported lifetime depression, and increased CVD risk. All *N*: 361 CBT *n*: 145 MBCT *n*: 144 Fitbit *n*: 72	100% self-report of lifetime depression	43.5 (11.5) years old **Male**: 17.5% **Ethnicity:** 7.2% Hispanic	**CBT:** digital (website and wearable) **MBCT:** digital (website and wearable) **Fitbit**: digital (wearable)	8 weeks	None	**CBT – Digital:** (i) ~30–40 min of materials for each week (ii) Fitbit **MCBT – Digital:** (iii) ~30–40 min of materials for each week (iv) Fitbit CBT and MCBT had similar materials which included client vignettes and videos **Fitbit – Digital:** (v) Just given a Fitbit
Young et al. (2017) United States	RCT 6 months	Adults currently prescribed antipsychotic medication and a BMI >30 or BMI >28 with weight gain of ⩾10 lbs in the last 3 months. All *N*: 276 WebMOVE *n*: 93 MOVE-SMI *n*: 95 C *n*: 88	SMI 100%	WebMOVE: 55.5 (9.2) years old MOVE-SMI: 53.8 (10.1) years old C: 54.2 (9.9) years old **Male:** WebMOVE: 95.8% MOVE-SMI: 92.3% C:97.7% **Ethnicity:** WebMOVE: 43.7% White 42.3% African-American MOVE-SMI: 8.5% White 41.0% African-American TAU: 37.5% White 51.1% African-American	**WebMOVE:** digital (website + weekly telephone calls) **MOVE-SMI**: in-person	6 months	TAU (educational handout on the benefits of weight loss)	WebMOVE* – **Digital**: (i) website with 30 interactive educational modules (15 on diet; 15 on physical activity) (ii) pedometer provided (iii) weekly telephonic peer coaching MOVE-SMI* – In-person: (iv) 24 × 60-min sessions (8 individual and 16 group) (v) utilises handouts, motivational techniques, visual learning aids, behavioural rehearsal, repetition, goal setting, homework, and diet plans. *Both offered the same educational content.

BD, bipolar disorder; BMI, body mass index; C, control; CBT, cognitive behavioural therapy; CVD, cardiovascular disorder; I, intervention; lbs, pounds; MCBT, mindfulness cognitive behavioural therapy; MH, mental health; mHealth, mobile health; PA, physical activity; RCT, randomised controlled trial; s.d., standard deviation; SMART, specific measurable attainable realistic and timely; SMI, severe mental illness; SZ, schizophrenia; SZ-AFF, schizoaffective treatment-as-usual (TAU); 1×/week, once a week; 2×/week, twice a week.

**Table 3.** Descriptive characteristics for the studies on digital interventions in SMI for others

Authors, year Country	Study design Study duration	Study focus	Target sample, *N*	Diagnosis (%)	Mean age (s.d.; range) Gender (male %) Ethnicity	Intervention mode	Number phases Intervention duration	Control condition/comparator	Detail about intervention
Hammond et al. (2020) United States	RCT 30 day post-discharge	Promoting seeking treatment for substance use after discharge	Self-reported substance use in last 30 days and a diagnosis of SUD and a psychiatric illness. All *N* = 95 C *n* = 47 I *n* = 48	MDD 42% BP 44% Other 14%	C: 39.8 years old (8.1) **I**: 38.5 years old (10.4) Male: C: 66% **I:** 68.8% **Ethnicity:** **C:** 57.4% White; 38.3% Black/African-American; 4.3% other **I:** 66.7% White; 33.3% Black/African-American	Digital (website)	Duration not stated but completed while an inpatient at specific, monitored times (up to 3 × 1 h sessions a week)	Control: TAU	65 interactive modules on substance use-related topics (e.g. drug refusal skills training effective, HIV and AIDS, and problem solving). Provided study laptops
Melamed et al. (2022) Canada	Single-arm, uncontrolled study 24 weeks	Perceived benefit of changing smoking habits, physical activity, and nutrition Secondary aim: change in behaviour	Aged 16–29, diagnosed with a psychotic disorder within the past 5 years. Al *N* = 70 HI *n* = 37 LI *n* = 33	SZ 13% SZ-AFF 11% BP 26% Depression with psychosis 7% Other 43%	HI: 22.7 years old (3.2) LI: 24.3 years old (2.9) Male: HI: 56% LI: 43% Ethnicity: 48% White	**HI:** digital (website and online calls) **LI**: digital (website and weekly reminder emails)	12 weeks	None	**HI:** (i) e-platform with educational modules about smoking, physical activity, and nutrition (ii) weekly one-on-one virtual sessions up to 60 min with health coach via the e-platform **LI:** (i) e-platform with educational modules about smoking, physical activity, and nutrition (ii) weekly automated reminder emails for the 12 weeks
Taylor et al. (2022) UK	Feasibility study 7 weeks	Sleep	Aged 16–65 with a schizophrenia-spectrum diagnosis and experiencing sleep difficulties for the least 4 weeks *N* = 14	MDD 42% BP 44% Other 14%	35.57 years old (10.88; 22–57) Male: 64.29% **Ethnicity:** 21.4% White; 42.9% Black/African/Caribbean/Black British; 14.3% Asian/Asian British; 14.3% mixed; 7.1% other	Blended – digital element (ExpiWell app)	6 weeks	Control: None	**In-person**: prior to the digital intervention, participants met with the research therapist to (i) set SMART goals (ii) guide them to modules relevant to their goals and struggles (iii) help them pick additional module **Digital**: uses CBTi techniques The ‘My Sleep Programme’ consisted of (i) six core weekly modules (30 min to complete) (ii) one participant-chosen module (‘Managing Worry’ or ‘Coping with Voices'). (iii) supportive feedback provided (not specified how)

BD, bipolar disorder; e-platform, electronic platform; FU, follow-up; HI, high intensity; IQR, inter-quartile range; LI, low intensity; MDD, major depressive disorder; NS, non-significant; OMI, other mental illness; s.e.m., standard error of mean; SUD, substance use disorder; SZ, schizophrenia; SZ-AFF, schizoaffective disorder; TAU, treatment-as-usual; TAU + TES, treatment-as-usual + therapeutic education system.

The HBC used included those delivered entirely digitally and those using ‘multi-component’ approaches (i.e. digital and in-person aspects). Tables 4–6 present study outcomes and interventions. Nineteen studies focused on smoking as the primary behavioural outcome (Tables 1 and 4). Fourteen studies focused on physical activity, weight loss, and cardio-metabolic health (Tables 2 and 5) and three papers focused on ‘other behaviours’ (Table 6), specifically sleep (Taylor, Bradley, & Cella, 2022), substance use (Hammond, Antoine, Stitzer, & Strain, 2020), and invoking changes in the perceived benefit of changing health behaviours, rather than the behaviour itself (Melamed et al., 2022). No HBC targeted sexual health.

**Table 4.** Key outcomes and findings from studies of digital interventions in SMI for smoking

Authors, year	Recruitment	Attrition	Adherence to intervention	Usage data	Usability (SUS) task scores	User perspectives and feedback (questionnaires)	User feedback (interview)	Behavioural outcomes	Physical and mental health outcomes
Browne et al. (2021)	NR	NR	NR	• Number of days interacted were similar between groups (LTQ: 34.1 days *v.* QuitGuide: 32.0 days) Those in LTQ interacted with app for more minutes per day (LTQ: 228.07 *v.* QuitGuide: 129.38) Those in LTQ had more interactions/clicks (LTQ: 335.5 *v.* QuitGuide: 212.7)	NR	NR	NR	Number of cigarettes smoked were reduced in both groups Mean change in cigarettes/day was higher for those in the LTQ [mean = 12.3 (11.5)] *v.* QuitGuide [mean = 5.9 (5.9)]	NR
Brunette et al. (2011)	43/48 approached (90%) were interested 41/48 (85%) enrolled Reasons not enrolled: 2 (4%) reading level 1 (2%) severe psychiatric symptoms 4 (8%) lack of interest or unstated reason	0% lost at 2 month FU	100%	NR	NR	NR	The electronic decision system was comprehensible, easy to use.	2 month FU: participants who used the decision support system were significantly more likely to have engaged in ⩾1 smoking cessation behaviour (67% *v.* 35%): attempted to quit (57% I *v.* 30% C) met with a specialist (38% I *v.* 0% C) started NRT (14% I *v.* 10% C) attended a CBT group (24% I *v.* 0% C) meet a clinician to discuss treatment (43% I *v.* 10% C)	NR
Brunette et al. (2012)	Not reported	0%	NR	NR	0 websites were usable for minimal computer users (<5 times) Casual users (⩾5 times) rating of ease to use: (1) pmusa.com (scored highest on the two tasks) (2) becomeannex.org (3) Smokefree.gov (4) whyquit.com	NR	NR	NR	NR
Brunette et al. (2016)	NR	NR	Phase 1: ⩾75% of users were highly satisfied with modules 2 and 4–8. Phase 2: 100% would recommend to friends 83% were very satisfied with the programme	Phase 2: averaged an hour (range 27–123 min), per session averaged 36 min (16–63 min) on each module. logged into the website an average of 7.5 (4–13) times over 3 weeks viewed an average of 1 document in the website library on average 15 (range 0–47) pages in the support group online forum were viewed. on average 2.3 posts (range 0–5) were posted in the support group			Phase 1: issues included: logging on navigating support groups finding and making new posts confusion if a page had two areas of content or function using mouse website readability. Phase 2: Improvements suggested: 1/3 said none 2 improve usability of support group 2 wanted more training	Phase 2 (self-reported at 3 weeks FU): 33% abstinence 17% no change in smoking (complete in office) 50% reduced smoking by half. Phase 1: NR	NR
Brunette et al. (2018)	89 consented and assessed for eligibility 81 (91%) were eligible and enrolled 7 (8%) screen fails 1 (1%; control) dropout	11.1% at 3 month FU	100% LTAS: 24% viewed additional video about NRT and bupropion 29% viewed additional video about varenicline	LTAS: on average 58 (±22) min on intervention Felt harder to understand compared	NR	A higher % of users rated LTAS at least good compared to users of NCI website (83.4% *v.* 71.4%) A higher % of NCI users felt it was ‘hard to understand’ (10.7% compared to 3.3% of LTAS users). LTAS: 87% liked the video host ‘a lot’. 53% liked the videos of doctors talking about cessation treatments. favourite part was the video host and the video quit stories. 20% said nothing could be improved. 23% wanted more info about health effects of smoking.	NR	LTAS participants significantly more likely to have quit smoking (~25% LTAS; ~9% NCI; ~4% control) Biologically verified at 3 months: 15% of LTAS participants, 0% control or NCI education More participants from LTAS (7.4%) had started any Verified treatment compared to those in NCI (4.3%) and control (4.5%) at 3-month FU	No adverse events reported
Brunette et al. (2019)	NR	5% attrition at assessment and 2 month follow-up	100% used intervention, 15% access intervention on phone 85% accessed intervention on computer 11/20 (55%) used NRT	Average sessions accessed 23.6 (s.d. = 17.05; range 10–48) The website was accessed on an average of 12.05 (s.d. = 10.57) days Average total minutes the website was used across the 8 weeks was 231.64 (s.d. = 227.13) min	NR	Participants were highly satisfied with the Website. 87% of the sessions were rated ⩾3 (4 = ‘I liked it very much’).	Positive feedback was received regarding the amount and content of information and ease of use To improve website: (i) improve audio quality (ii) less repetitive (iii) functionality on phones (iv) access website for >8 weeks	25% reduced their smoking 10% biologically verified abstinence Mean cigarettes per day reduced (NS) at 8 weeks, from 25.6 to 20.3	NR
Brunette et al. (2020)	184 consented 162/184 (88%) were randomised to interventions 11/184 (6%) were ineligible 1 moved 1 lost to FU 1 unable to continue 8 (4%)declined (reasons not stated)	9.9% (*n* = 16) at 3 months 10.5% (*n* = 17) at 6 months	100%	NR	LTAS usability and satisfaction mean summary index scores were significantly higher compared with those assigned to NCI	NR	Most participants (95.38% of LTAS users and 83.1% of NCI education users) reported they were ⩽least satisfied 97% would recommend their respective intervention to a friend.	Biologically verified abstinence at 6 months: 1% of LTAS participant 8% NCI education More participants from NCI had started any cessation treatment compared to LTAS (46% *v.* 32%; NS) 17.9% of LTAS and NCI participants reported ⩾7 days of self-reported abstinence 4.3% (7/162) of total participants had biologically verified 7-day PPA at 6-month FU	No adverse events were reported during the use of the interventions.
Ferron et al. (2011)	6/89 (7.9%) referred did not respond 7/89 (7.9%) was not interested 4/89 (4.5%) DNA research visit 2/89 (2.2%) ineligible due to their reading ability 71/ 89 (89.8%) consented	NR	NR	NR	NR	NR	Barriers to using the first iteration included: not knowing how to use a mouse hand tremors (Parkinson-like symptoms) lack of familiarity with computer interfaces Participants agreed that the information was easy to understand and that the programme was easy to use.	NR	NR
Gowarty et al. (2021a)	19% (*n* = 19/98) screened consented 17% (*n* = 17/98) included in the study (2 were ineligible due to carbon monoxide levels) 35% (*n* = 34) were ineligible 46% declined Reasons for refusal: 7% due to work/childcare commitments 1% due to moving 10% (*n* = 10) no phone 28% no reason	0% at visits 1 and 2	NR	Usage: quitSTART was used on average for more days over 2 weeks [10.8 (s.d. 3.5) *v.* QuitGuide [4.6 days (s.d. 2.8)] quitSTART [41 (s.d. 26)] had more interactions compared with QuitGuide [5.6 (s.d. 3.8)] For both apps, those with SMI–PTSD interacted for more days and had a greater number of interactions than those with psychosis.	Usability (SUS): QuitGuide score higher than quitSTART at both visits Diagnosis seemed to influence SUS score for QuitGuide, with participants with psychosis scoring higher and quitSTART with those with psychosis SUS low at visit 1 but increasing to an acceptable level at visit 2	NR	Task completion: QuitGuide completion rates were high at both visits and similar between diagnosis groups quitSTART completion rates were lower at visit 1 (especially for those with psychosis) but similar to QuitGuide at visit 2.	77% reported attempting quit/reduce smoking during the 2-week trial period. The % of people who attempted to quit/reduce smoking similar for each app (78% QuitGuide *v.* 75% quitSTART users). 2 quitSTART participants no longer smoked at visit 2 (confirmed with breath carbon monoxide <7 ppm)	NR
Gowarty et al. (2021b)	98 screened, 19% (*n* = 19) screened consented 35% (*n* = 34) were ineligible 28% (*n* = 27) declined, no reason 7% refused due to work/childcare commitments 1% moving 10% (*n* = 10) no phone 2/19 who consented were ineligible due to carbon monoxide levels 17% (*n* = 17) included in the study	Attrition: 0% at visits 1 and 2	100% (17) completed both visits 1 and 2 Usage data available for 88% (15)	Days used: quitSTART was used on average for more days over 2 weeks [10.8 (s.d. 3.5) *v.* QuitGuide [4.6 days (s.d. 2.8)] **App interactions:** quitSTART [41 (s.d. 26)] had more interactions compared with QuitGuide [5.6 (s.d. 3.8)] **Median responses to notifications:** quitSTART participants responded more to notifications 18.5 (range 0–37) compared to those on QuitGuide 1 (range 0–8)	**SUS scores:** QuitGuide's scores were higher at visit 1 and similar at visit 2 when compared to quitSTART's scores. QuitGuide SUS visits 1 and 2 (64; range 30–77.5, s.d. 18 and 66; range 25–85, s.d. 18, respectively). quitSTART SUS increased from visit 1 (55; range 25–82.5, s.d. 20) to visit 2 (64; range 35–85, s.d. 16).	**Ease of use** QuitGuide higher at both visits. Overall satisfaction: QuitGuide 78% at both visits; quitSTART visit 1 50%, visit 2 75%. **Task completion:** All 9 tasks were completed by ⩾75% of QuitGuide participants 8/9 tasks were completed by ⩾75% of quitSTART participants	QuitGuide was easy to use	Similar quitting rates for both groups (78% quitGuide and 75% quitSTART)	NR
Heffner et al. (2018)	NR	NR	NR	ACT arm, the BD group (*M* = 13.5, s.d. = 68.1) had a higher number of logins compared to other two. There were no differences in the number of logins to the Smokefree.gov website by diagnostic group.	NR	NR	NR	6-month PPA (NS between groups): total 20%; no MHC 22%; BD 14%; AD 18%, 12-month PPA (NS between groups): total 22%; no MHC 25%; BD 16%; AD 18%	NR
Heffner et al. (2020)	51/119 (42.9%) enrolled over 24 months 22/119 (18.5%) not eligible 5/119 (4.2%) not interested 11/119 (9.2%) unable to attend 8/119 (6.7%) missed the window 7/119 (5.8%) don't smoke enough 15/119 (12.6%) other reasons	14/51 (27%) end of treatment 10/51 (20%) at 1-month FU	NR	Days logged in nearly twice as high for Smokefree.gov Mean number of logins was twice as high for WebQuit Plus (10.3 *v.* 5.3)	NR	NR	NR	A greater % of WebQuit plus (12%) participants had 7-day PPA at end of treatment compared to Smokefree.gov (8%). 7 day PPA at 1 month FU was 8% for both groups	Depression and mania score slightly improved (NS) for Smokefree.gov Depression no change and mania score slightly improved (NS) for WebQuit 3 AEs were coded as ‘possibly related’, and 2 AEs were coded as ‘probably related to the intervention’. 1 SAE was judged to be possibly related to intervention (1 WebQuit Plus participant had increased impulsivity and suicidal ideation).
Klein et al. (2019)	NR	**0%**	NR	NR	NR	83% were enthusiastic about engaging in a social network and liked chatrooms to connect to others.	Personalising app to users' psychosocial needs (i.e. stigma and social isolation) Smoking helps them cope with mental illness. Smoking increased when distressed, so app should be adapted to support this. App should normalise smoking relapses. App could assist quitting smoking in real time. App interface needs to be simple. Participants liked the crave profile generated in the app, and perceived smoke logging to be useful.	NR	NR
Medenblik et al. (2020)	35/42 (83.3%) screened were enrolled	NR	Only 2.9% (1) used NRT offered, as they were reluctant to add another prescription to medication regime	NR	NR	NR	NR	iCOMMIT: 9.5% self-reported abstinence at 7 and 30 day FU. 14.3% were abstinent (verified by saliva) at 6 months ITC: 15.4% self-reported abstinence at 7 and 30 day FU. 15.4% were abstinent (verified by saliva) at 6 months	NR
Vilardaga et al. (2016)	5/10 (50%) participated 30% were Ineligible 20% lost at contact	0%	100% participants tracked their cigarettes on a daily basis	NR	Average SUS score was 65.5 (s.d. = 18.6), 5 below the industry standard	NR	• Incremental rewards better than setting larger saving goals. • More focus on the process of cutting down rather than quitting. • QuitPal served as an awareness tool, which needed finer-grained cigarette tracking. • The need for interactive and motivating features. • Multiple layers of app made it difficult for users to interact.	NR	NR
Vilardaga et al. (2018)	NR	0%	100%	NR	SUS learnability (mean = 60) SUS usability (mean = 80) SUS total (mean = 74)	NR	• Homescreen confusing • Liked the gamification (quizzes and rewards badges) • Liked the cartoons and storytelling • Liked Acceptance and commitment therapy • Liked simplicity of app • Need for further technical support	NR	NR
Vilardaga et al. (2019)	37% (14/38) screened positive for 24% (9/38) were eligible 18% (7/38) completed the study	22.2% (2//9) 1 due to hospitalisation 1 whose phone was stolen	NR	Mean days across participants LTQ 90%; QG 59% Mean minutes across participants: LTQ: 14 min 45 s QG: 6 min 50 s	(P1 and P2): LTQ's usability scores were above the standard cut-off (i.e. SUS = 68) in both cases , with QG slightly outperforming LTQ in P1, but largely underperforming LTQ in P2.	Low levels of UE for both participants with each of the app's tracking features (e.g. cigarette use, mood, craving), with a larger average for the QG app.	Bi-phasic AB single-case studies (P5, P6, and P7): **LTQ**: high retention of smoking cessation content engagement through gamification (quizzes) simple to use positive emotional experience **QG**: app and features difficult to navigate track and charts for monitoring were perceived useful app not very engaging, very serious mixed agreement about usefulness of app features	NR	NR
Vilardaga et al. (2020)	498 potential candidates 11% (55/498) refused 25.7% (128/498) ineligible 31.1% (155/498) unconfirmed 2% (12/498) unsure 14.1% (70/498) lost before randomisation 1% (2) self-withdraw before randomised 18.5% (92/498) in-person screening 12.7% (63/498) randomised	4 week FU 8% (5/63); 8 week FU 14% (9/63); 12 week FU 9% (6/63); 16 week FU 3% (2/64)	NR	Similar number of days of app use for both apps (LTQ 34; QG 32) LTQ significantly more app interactions than QuitGuide (335 *v.* 205; *p* = 0.001) LTQ significantly longer durations of app use compared with QG (4.24 h. *v.* 2.14 h; *p* = 0.044) LTQ higher usability scores than QG (85 *v.* 79, *p* = 0.046)	NR	NR	NR	NS difference between 2 groups abstinence (biochemically verified) at FU. Seven-day PPA rates at week 4 favoured QuitGuide, with an 8% abstinence difference compared to LTQ However, in the remaining follow-up assessments (weeks 8, 12, and 16), abstinence rates were higher in the LTQ *v.* the QuitGuide arm QuitGuide participants made statistically significant more quit attempts compared to LTQ participants At week 16 FU, LTQ lead to greater reductions in **CPD** (12.3 *v.* 5.9 for QuitGuide; *p* = 0.010), but NS when adjusted for baseline CPD	Small reductions in symptom severity, anxiety, and depression for all. Increases in positive symptoms and a statistically significant increase in negative symptoms for all. LTQ arm had a mean of 1.12 (s.d. 1.14) intervention (‘related’ or ‘possibly related’ AEs) per participant QuitGuide group had a mean of 1.24 (s.d. = 1.38) intervention (‘related’ or ‘possibly related’ AEs) per participant
Wilson et al. (2019)	NR	Cohort 1: 28.6% (*n* = 2) withdrew prior to study 20% (1/5) attrition at 3 month FU Cohort 2: 2/13 (15.4%) withdrew prior to study 12.5% (1/8) attrition at 3 month FU	Cohort 1: 40% (*n* = 2) had high adherence to both therapy sessions and carbon monoxide readings Cohort 2: 63% (*n* = 5) had high adherence to both therapy sessions and carbon monoxide readings	NR	NR	NR	Counselling sessions were the best part of the treatment mCM was acceptable but some found it difficult to film and upload their carbon monoxide readings. Cohort 1: 20% felt the stay quit app was useful	Cohort 1: 40% reported abstinence. 1 participant (who smoked 30 cigarettes per day) failed to reduce his smoking Cohort 2: 3 participants (38%) reported abstinence 1 participant (who did not complete any sessions) failed to reduce smoking	NR

AEs, adverse events; CPD, cigarettes per day; LTAS, Lets Talk About Smoking; LTQ, learn to quit; MH, mental health; NCI, National Cancer Institute; NS, NOT SIGNIFICANT; PNTS, prefer not to say; PPA, point prevalence abstinence; QG, quit guide; RCT, randomised controlled trial; SAEs, serious adverse events; s.d., standard deviation; SMI, severe mental illness; SUS, System Usability Scale; TAU, treatment-as-usual.

**Table 5.** Key outcomes and findings from studies of digital interventions in SMI for physical activity

Authors, year	Recruitment	Attrition	Adherence to intervention	Usage data	Usability (SUS)	User perspectives and feedback (questionnaires)	User feedback (interview)	Behavioural outcomes	Physical and mental health outcomes
Aschbrenner et al. (2015)	10/24 (42%) participated Reasons for refusal/exclusion: not interested (*n* = 10; 42%) excluded due to BMI (*n* = 2; 8%) moved (*n* = 1; 4%) lost at FU (*n* = 1; 4%)	1/10 (10%) due to unrelated health concerns.	Phase 1 – training 100% Phase 2: 79.9% attended weekly 1-on-1 meeting with peer health coach 81% attended weekly 1-on-1 with fitness trainer 69.5% attended weekly group sessions. Phase 3: 77.8% attended biweekly health coach meetings. 57.8% attended weekly nutrition group sessions. 28.6% attended weekly exercise group sessions.	NR	NR	Participants: perceived the programme as beneficial, useful, and convenient would recommend the programme to a friend 67% reported Fitbits were convenient to help them reach their set goals	Peer interaction was perceived as the best part, particularly peer health coaches and learning from others experiences Barriers included: schedule conflicts and concerns about their physical limitations	Stake holders interviews: participants reported to health coaches a reduction in high-calorie snacks and sugary beverages participants reported beginning/increasing exercise levels	56% of participants lost weight (not significant change) mean weight loss 2.7 ± 2.1 kg
Aschbrenner et al. (2016a)	13/29 (45%) participated 8 not interested (27.5%) 8 not eligible (27.5%) Reasons for exclusion: 2 had transport issues 2 enrolled in another weight loss study 2 due to medical reasons 1 due to BMI 1 moved	2/13 (15%) at 6 months 1 withdrew due to family and work commitments 1 completed baseline but never attended any sessions	100% used Fitbit 76% used Facebook group 28% of optional exercise sessions were attended 11.2/40(s.d. = 12.8). Mean number of weight management sessions attended was 16/24	NR	NR	NR	NR	NR	72% lost weight 28% achieved clinical significant weight loss Mean weight loss of 7.76 (±12.4 pounds) BMI decreased significantly (mean decrease of 1.25 ± 1.99 kg/m^2^) 17% showed clinically significant improvements in cardiovascular fitness
Aschbrenner et al. (2016b)	13/29 (45%) participated 8 not interested (27.5%) 8 not eligible (27.5%) Reasons for exclusion: 2 had transport issues 2 enrolled in another weight loss study 2 due to medical reasons 1 due to BMI 1 moved	15% (*N* = 2) at 6 months	Overall attendance rate was 56% Attendance rate high for first 12 weeks (79%) Attendance rates dropped to 33% for weeks 13–24 Average number of weight management sessions attended was 13.5/24 (s.d. 4.6) Attendance rate for the optional group exercise sessions was 28%, with an average of 11.2 (s.d. = 12.8) sessions	NR		73% agreed programme helped 91% were satisfied with the programme 91% found it useful and easy to understand 91% would recommend to a friend 81% felt programme meet their needs	Food diary helpful for portion control Dietary tracking was complex Participants felt the focus should be on balanced meals not counting calories Group exercises were fun and motivating Weekly weigh ins were demotivating Programme needs to be more active and engaging Wanted more peer interaction Wanted grocery lists Cooking skills would have been helpful Wanted more training on Facebook and Fitbit app	NR	45% of participants were below their baseline weight No overall significant changes in mean weight or fitness 45% improved their cardiorespiratory fitness 36% of participants met the criteria for clinically significant cardiovascular risk reduction
Aschbrenner et al. (2021)	NR	17% (*N* = 26) did not compete FU at 6 months 31% (*N* = 46) did not compete FU at 12 months No differential attrition between 2 groups	**PeerFIT:** 1st phase: mean lifestyle sessions attended was 6.36 (6.9) out of 24 mean exercise sessions attended was 5.56 (6.5) out of 24 75% of participants attended ⩾1 lifestyle session 70% of participants attended ⩾1 exercise session 2nd phase: mean exercise sessions attended was 1.86 (s.d. 3.2) out of 24 (0.1%). 42% of participants attended ⩾1 exercise sessions 66% used the Facebook group 78% used text messaging BEAT: mean telephone coaching sessions attended was 4.26 (s.d. 1.8) of six offered. 97% attended at ⩾1 coaching session. 91% used text messaging	NR	NR	NR	NR	NR	**6 months FU:** 52% of PeerFIT and 58% of BEAT participants were ⩽ their base weight 29% of PeerFIT and 29% of BEAT participants achieved clinically significant reduction in CVD risk **12 months FU:** 50% of PeerFIT and 54% of BEAT participants were ⩽ their base weight 25% of PeerFIT and 30% of BEAT participants achieved clinically significant reduction in CVD risk Both FU time points: Participants achieved clinically and statically significant weight loss (no significant between-groups) No significant between-group difference for weight-loss CVD risk No significant between-group difference for CVD outcomes
Campos et al. (2015)	8/46 not eligible 6/46 declined (no reasons provided) 69.6% of those approached and 84.2% who were eligible consented	3/32 (9.4%) all from intervention group	69.3% completed the intervention within the proposed timescale.	Average level of games completed was of 45.54 ± 12.18 84.62% completed more than half of the projected levels	NR	92% rated the exergames intervention and interactive. Over 90% reported an increase in at least 1 intervention effect [endurance (76.9%), motor functions (53.8%)]. 76.9% agreed the intervention promoted a healthier lifestyle and it is acceptable alternative to perform PA. 76.9% were satisfied with game control/function, 84.6% would not use this type of equipment without technical assistance 84.6% felt confident in their performance in the game. 7.7% did not agree with session duration and intervention length 53% agreed it was easy to learn to use the game.	NR	NR	No significant multivariate effects for combined physical activity and motor function outcomes No significant differences found for PANNS
Looijmans et al. (2019)	I: Consented: 140 (82.4%) of the 170 recruited *n* = 8 no longer interested *n* = 8 logistic reasons *n* = 14 consent not given C: Consented: 104 (91.2%) of the 114 recruited *n* = 5 no longer interested *n* = 3 logistic reasons *n* = 1 consent not given *n* = 1 surgery	I: 43.4% (*n* = 57) at 6 months; 33.1% (*n* = 43) at 12 months C: 43.4% (*n* = 57) at 6 months; 33.1% (*n* = 43) at 12 months	108/140 (77%) completed at ⩾1 lifestyle behaviour screening and made lifestyle plans with lifestyle goals. 12% were low users and (*N* = 13) had no FU reports 56% were medium users (*N* = 60) and had medians of 4.0 [2.3; 7.0] reports. 32% were high users (*N* = 35) and had 14.0 [11.0; 18.0] FU reports.	NR	NR	NR	NR	NR	Waist circumference change (after adjusting for antipsychotic medication) between intervention and control were −0.15 cm (NS) and −1.03 cm (NS) after 6 and 12 months, respectively NS differences for BMI and MS *Z*-score
Macias et al. (2015)	11 meet eligibility criteria	18.2% (2/11) 10% at 4 week FU	98% responded to personalised text messages 39% adhered to daily walk prompts 70% participants achieved ⩾2 walks per week (range 8–26, total walks over a 4-week period) Walks averaged 15–36 min per walk. Reasons for not walking included psychiatric distress to competing sports activities	NR	The app was used for 94% of days in the study All participants used WellWave for 85% or more of their days in the study 73% responded to prompts (mean 101.8 response overall and 3.54 per day) 50% used the app every day. Mean days app used was 26.8 (s.d. 2.04)	100% of participants were satisfied with the app overall.	Favourite features were text message conversations with peer staff, and peer staff testimonial videos Complaints: (i) colour/sound of app (ii) the study too short. Improvements: (i) more peer videos (ii) one person suggested more interaction with other app users	70% of participants achieved ⩾2 walks per week	Slight improvement in physical health self-ratings from pre-test to post-test
Muralidharan et al. (2018)	NR	**WebMOVE:** 25% (*n* = 23) at 3 months; 17.4% (*n* = 16) at 6 months **MOVE-SMI:** 16% (*n* = 15) at 3 months; 13.8% (*n* = 13) at 6 months **TAU:** 23% (*n* = 20) at 3 months; 14.9% (*n* = 13) at 6 months	WebMOVE: 18% (*n* = 17) did not attend MOVE-SMI: 23% (*n* = 22) did not attend	NR	WebMOVE participants completed slightly more modules. WebMOVE: 14.7 (±12.2) modules completed out of 30 (49%) MOVE-SMI 9.7 (±6.2) modules completed out of 24 (40.4%)	NR	Walking was used as the primary source of exercise in WebMOVE and MOVE-SMI. Walking was perceived as manageable and low impact. The social aspect of exercise was given as most people's motivation to exercise. Barriers to PA: (i) motivation and time (ii) physical limitations or disability (iii) pain (they needed to modify exercise) Peer coaches were perceived crucial in the interventions. Participants liked that peer coaches reminded them of weekly goals, kept them accountable, motivated them, and provided support. WebMOVE participants found the pedometer helped them with goal setting and improved their motivation.	WebMOVE: When compared to usual care, there was a significant group difference for total physical activity at 6 months (*p* = 0.044), and WebMOVE minutes spent walking at 3 and 6 months, but NS. WebMOVE baseline 658 min (s.d. 931); 3 months 839 min (s.d. 1108); 6 months 748 min (979) MOVE-SMI: Significant increase in minutes spent walking at 3 and 6 months Baseline 443 min (s.d. 516); 3 months 641 min (s.d. 899); 6 months 500 min (493)	NR
Naslund et al. (2015a, 2015b)	NR	1/10 (10%) due to mental health	Ten wore the devices for a mean of 89% (s.d. 13%) of the days in the study. Five participants wore the devices 100% of the time.	NR	NR	Participants reported high satisfaction	(i) Wearable were easy to use (ii) Helpful for setting goals, motivating, and useful for self-monitoring (iii) Frustration when they forgot to sync steps or wear it (iv) Wearables were perceived as expensive (v) They felt wearables would not be affordable for low-income individuals Participants liked the rewards and animation for wearables. No privacy concerns were reported	NR	NR
Naslund et al. (2016)	Recruitment: 13 were recruited 2 dropped out prior due to scheduling conflicts	23% (*n* = 3) due to medical or personal reasons	82% of participants used their Fitbit Zips for over 80% of the days enrolled. Reasons for not wearing Fitbit: device malfunction dead battery temporarily misplaced lost the device, forgot to put it on	NR	Fitbits were worn for an average of 84.7% (s.d. = 18.1%) days	Fitbit easy to use, highly satisfied with the device. Fitbit helped them to be more active, and that it was fun and motivating to use. Participates would recommend it to a friend. Participants indicated they would like to continue to use a Fitbit in future.	Feedback included: (i) Fitbit increased awareness of their PA (ii) Useful for setting goals (iii) Calories burnt feature was not helpful (iv) Technical difficulties with using app and/or phone (particularly for those with limited experience) (v) No issues with Fitbit itself	NR	NR
Naslund et al. (2018)	32 enrolled 25/32 participated (78%)	1/32 (3%) stopped due to substance use; 1/32 (3%) pregnancy; 1/32 (3%) hospitalisation; 2/32 (6%) not interested; 2/32 (6%) lost at FU	76% (*n* = 19) joined the Facebook group. Reasons for not joining the Facebook group: (i) not interested (ii) finding it too difficult to use (iii) preference for face-to-face interaction (iv) only 1 had concerns about privacy. Attendance was higher at the weekly in-person group weight management sessions for those who joined the Facebook group (mean = 17.3 sessions, s.d. = 7.3 *v.* those who didn't mean = 14.6 sessions, s.d. = 6.6; NS)	NR	Overall there were 208 interactions with an average of 13.0 (s.d. = 17.2) interactions per participant	NR	NR	NR	Those who interacted in the Facebook group had clinically significant reduction in cardiovascular risk (⩾5% weight loss) or improved fitness (Facebook group 19.1 ± 20.5; not in group 3.9 ± 6.7)
Olmos-Ochoa et al. (2019)	277/1429 (19%) screened were randomised to a group	NR	WebMOVE 35% (about 10.5 sessions) sessions were completed MOVE-SMI 31% (about 5 sessions) sessions were completed Several participants misplaced or broke their pedometers.	NR	NR	NR	Participants were satisfied with their intervention Barrier to healthy eating: (i) lack of control over food preparation Barrier to physical activity: (i) physical problems (ii) time, money, environment, and safety problems (iii) lack of motivation and support from others Barriers to intervention: (i) financial hardship (ii) lack of reliable (iii) housing and transportation (iv) comorbid physical and mental health issues (v) not enough time (vi) technology issues WebMOVE feedback: (i) issues signing-on but were able to after help from their peer-coaches (ii) technical issues with the pedometers and logging their daily steps (iii) forgetting to wear the pedometer daily MOVE-SMI: (i) difficulty interacting with others and meeting new people	NR	NR
Sylvia et al. (2021)	Contacted (*n* = 5500) (MoodNetwork) and 4.1% emailed back. *n* = 70 457 (Health-eHeart) contacted and 11.5% emailed back. 361/70 957 (1%) participated	NR	CBT arm: 15/145 (10%) missing Fitbit data 84/145 (58%) did not complete all e-visits MBCT arm: 21/144 (10%) missing Fitbit data 91/144 (62%) did not complete all e-visits Fitbit arm: 9/72 (12.5%) missing Fitbit data 44/72 (61%) did not complete all e-visits	NR	NR	NR	NR	NR	NR
Young et al. (2017)	1429 were screened for eligibility 19.3% (276) were enrolled and randomised 58.3% did not meet inclusion criteria (*n* = 833) 16.7% declined to participate (*n* = 239) 1.2% enrolled in other study (*n* = 17) 1.1% not receiving treatment (*n* = 16) 3.4% clinician did not approve of research (*n* = 48)	Total: 39/276 (14.1%) WebMOVE: 22/93 (23.7%) MOVE-SMI: 17/95 (17.9%)	100% completion of intervention component	NR	WebMOVE: 49% modules completed average sessions completed 14.7 (s.d. = 12.2) MOVE-SMI: 41% modules completed average sessions completed 9.7 (s.d. = 6.2)	NR	WebMOVE: Overall, the intervention was well received. Feedback included: (i) helpfulness of educational modules and pedometer (ii) positive reaction to the peer coaches Less prevalent themes were difficulty in accessing a computer and the desire for a walking group MOVE-SMI: The intervention received a mixed response. Feedback included: (i) participants' discomfort with a group format (ii) transportation was a barrier	NR	WebMOVE change in BMI 34.9 ± 0.43 to 34.1 ± 0.43 No substantial change in BMI was seen in the MOVE-SMI group, or usual care group Participants in the WebMOVE group were more likely to lose 5% or more of body weight compared to those in the MOVE-SMI group (*p* = 0.01) 37% of WebMOVE participants lost 5% or more of body weight at 6 months 18% of MOVE-SMI participants and 25% of the usual care group lost 5% of weight by 6 months

BMI, body mass index; C, control; CBT, cognitive behavioural therapy; CVD, cardiovascular disorder; I, intervention; lbs, pounds; MCBT, mindfulness cognitive behavioural therapy; MH, mental health; mHealth, mobile health; PA, physical activity; RCT, randomised controlled trial; s.d., standard deviation; SMART, specific measurable attainable realistic and timely; SMI, severe mental illness; SUS, system usability scale; TAU, treatment-as-usual; 1×/week, once a week; 2×/week, twice a week.

**Table 6.** Key outcomes and findings from studies of digital interventions in SMI for others

Authors, year	Recruitment	Attrition	Adherence to intervention	Usage data	Usability (SUS)	User perspectives and feedback (questionnaires)	User feedback (interview)	Behavioural outcomes	Physical and mental health outcomes
Hammond et al. (2020)	95/213 (44.6%) enrolled 81/213 (38%) were not eligible 17/213 (8%) not interested 20/213 (9.4%) excluded other reasons	Baseline: 9/95 (9%) higher attrition for intervention group (15% *v.* 4%) 30 day post-discharge: 9/95 (9%) similar rates of attrition for the 2 groups	TAU + TES: 58% completed >2 modules	On average 5.5 (s.e.m. = 0.8; range 2–23) modules were completed	NR	TES group reported significantly more usefulness for OMI (TAU: *M* = 7.9 *v.* TAU + TES *M* = 8.8) Usefulness of the hospital treatment: rated similar between groups Usefulness of the hospital stay for SUD problems rated similar between groups TAU + TES: overall usefulness Ease of understanding Interest in topics and satisfaction were rated high (7.8–9.2)	NR	Similar rates of enrolment between two groups. TAU: 54% enrolled in SUD treatment TAU + TES: 56% enrolled in SUD treatment	NR
Melamed et al. (2022)	192/510 (37.6%) were eligible 70/510 (13.72%) were recruited 9.7% of participants ineligible as they had not been on stable medication dose for 4 weeks 25.5% unable to contact 17.3% not interested	Condition: 4 (5.7%) lost (2 from each) Reasons for attrition: 2 were lost at FU 1 time commitment 1 no reason Attrition at 24 weeks: LI: 60% (*n* = 14) HI: 59% (*n* = 17)	HI group: 62% completed ⩾6/12 weekly calls 21% completed ⩽10 weekly calls	A>% of HI participants used the e-platform for ⩾10 days compared to those in LI (52% *v.* 21%) Higher total hours of use in the HI (mean = 7.6) compared to LI (mean = 3.6) 48% of HI logged on the e-platform for 5 or more hours *v.* 21% from LI	NR	NR	NR	No changes in health behaviours over time	NR
Taylor et al. (2022)	21/60 (35%) referrals were screened 14/21 (66%) of those screened consented 13 (21.7%) started the intervention 5 (8.3%) were ineligible 2 (3.3%) not interested	2 lost (1.4%) Reasons for attrition: 1 due to technical issue 1 was admitted to hospital	53.8% engaged with all 7 modules 100% engaged with at least 2 modules Engagement declined with time, from 100% at week 1 to 61.5% by week 6 Managing worry (*n* = 11; 84.6%) was the most selected optional module	Mean number of modules engaged with was 5.6 (s.d. = 1.8)	NR	Acceptability criteria were not met for two participants (1 due to disruption to lives and 1 due to usual daily activities): two-thirds met acceptability criteria in terms of ease of remembering to use 81.8% considered the app enjoyable	App was useful Users had positive experience App easy and convenient to use Access to therapist support was advantageous Barriers included: technical issues accessing app/internet difficulty navigating app Improvements: gamification and variety of content was desired duration longer	45.5% no longer scored above cut-off for insomnia disorder Large effect size on sleep [insomnia severity *d* = 1.02 and sleep quality (PSQI) *d* = 0.83]	Small-to-medium effect size on mental health outcomes (depression *d* = 0.42; anxiety *d* = 0.35, stress *d* = 0.24)

e-platform, electronic platform; FU, follow-up; HI, high intensity; IQR, inter-quartile range; LI, low intensity; OMI, other mental illness; NR, not reported; NS, not significant; s.e.m., standard error of mean; SUD, substance use disorder; TAU, treatment-as-usual; TAU + TES, treatment-as-usual + therapeutic education system.

### Overview of included studies

Results of the included studies were synthesised in their respective classes of health behaviours, namely: (i) smoking; (ii) physical activity, weight loss, and cardio-metabolic health; and (iii) other behaviours. For each HBC class, feasibility, acceptability, usability, and impacts on behaviour/outcomes were summarised.

#### Smoking

Eight studies delivered HBC through smartphone apps and nine used web-based interventions. The remaining two were multi-component interventions (Table 1).

*Feasibility.* Recruitment rates ranged from 13% to 91% across studies (Table 4). Overall, attrition was low, ranging from 0% to 23%; common reasons for dropout included hospitalisation and loss of interest. Adherence to digital interventions was generally high, ranging from 43% to 100% (Table 4).

*Acceptability.* Apps developed for those with an SMI had greater engagement when compared with apps for the general population (Browne, Halverson, & Vilardaga, 2021; Vilardaga et al., 2019, 2020). One website, Lets Talk About Smoking (LTAS), was developed specifically for individuals with SMI, providing interactive tailored smoking cessation advice. This scored more highly on patient satisfaction when compared to users of static National Cancer Institute (NCI) patient education handout, which was developed for the general population (Brunette et al., 2018).

Participants reported links between symptom severity and smoking, and saw benefits in tracking smoking alongside their mental health (Klein, Lawn, Tsourtos, & van Agteren, 2019). Real-time support, such as a person or distraction task, was deemed essential to help with cravings (Klein et al., 2019).

*Usability.* People with SMI viewed easy navigation and engaging content and design as preferable, or even essential, for digital HBC (Brunette et al., 2011; Klein et al., 2019; Vilardaga et al., 2016, 2018). Issues were reported with readability, difficulty using support chatrooms, and navigation for certain websites or apps, particularly if pages had multiple functions. Difficulties simultaneously filming and uploading carbon monoxide readings were also reported (Wilson et al., 2019). In Brunette et al.'s (2012) study, four websites developed for the general population were difficult to use among people with SMI who had less experience using computers. Promisingly all participants learnt to use the website developed specifically for SMI populations, regardless of experience, with minimal training (one to three sessions) (Brunette, Ferron, Gottlieb, Devitt, & Rotondi, 2016).

Several apps developed for the general population (QuitGuide, quitSTART, QuitPal) scored below acceptable standards on the ‘System Usability Scale’ in some studies. In Vilardaga et al.'s (2019) study, both QuitGuide and the ‘Learn to Quit’ (LTQ) app – developed for people with SMI – met usability cut-offs as rated by two (all) participants. It is worth noting that when detecting usability problems, studies using samples as small as five can be deemed acceptable (Lewis, 1994).

*Behaviour and health outcomes.* All 13 studies, which evaluated digital HBCs' impact on smoking behaviours, found self-reported smoking reductions (Table 4). Five studies confirmed smoking abstinence through biochemical verification (Table 4). Unpromisingly, in one intervention, which took a multi-component approach, self-reported 7-day point prevalence abstinence decreased from 38–40% (from both cohorts) to 9.4% in the pilot RCT (Medenblik et al., 2020; Wilson et al., 2019).

Notably, Brunette et al. (2018) found, after 3 months, greater percentage of LTAS participants had biologically verified abstinence, compared to the NCI education group. Additionally another app developed for people with SMI (LTQ) was more effective in promoting smoking cessation, with those assigned to the QuitGuide app (developed for the general population) making more quit attempts and subsequently more relapses (Browne et al., 2021; Vilardaga et al., 2020).

Only two studies measured mental health outcomes. Heffner et al. (2020) reported a potential improvement in depression and mania scores, while Vilardaga et al. (2020) demonstrated a reduction in negative symptoms and a small non-significant reduction in depression, anxiety, and symptom severity across both groups.

#### Physical activity, weight loss, and cardio-metabolic health

One study delivered HBC through a smartphone app (WellWave), two studies used a web-based intervention and one study used an app with an associated wearable device. Seven studies used a multi-component approach (Table 2). Three studies compared web-based interventions with in-person interventions (Muralidharan et al., 2018; Olmos-Ochoa et al., 2019; Young et al., 2017).

*Feasibility.* Recruitment rates varied across studies, with studies recruiting from veteran centres reporting lower rates (19% participated and 58% were ineligible) than studies recruiting from mental healthcare services (42–45% participated and 28% were ineligible; Table 5). The highest recruitment rates were observed in studies conducted through outpatient clinics with subsequent participation not involving additional in-person sessions (Campos et al., 2015; Looijmans, Jörg, Bruggeman, Schoevers, & Corpeleijn, 2019).

Where reported, overall retention was high, with 75–90% of participant completing follow-up measures at the final time point, which ranged from 1 to 6 months (Table 5). Retention rates dropped after 12 months, to around 33% (Aschbrenner et al., 2021; Looijmans et al., 2019). Reasons for dropout included health concerns, hospitalisation, and competing time commitment.

Levels of adherence were generally high, particularly with digital components of the studies (Table 5). In one study of 32 participants, the in-person exercise sessions achieved only a 28% attendance rate, while 100% and 76% used the provided Fitbit and private Facebook group, respectively (Aschbrenner, Naslund, Shevenell, Kinney, & Bartels, 2016a). Similar findings were reported in Aschbrenner et al.'s (2021) study, with 70% of PeerFit participants attending at least one in-person exercise session, while 97% of BEAT participants attended at least one online coaching session (Aschbrenner et al., 2021).

*Acceptability.* Usage of digital interventions was also generally high (Table 5); in Muralidharan et al. (2018), Olmos-Ochoa et al. (2019), and Young et al. (2017), more modules were completed by those in the digital *v.* the in-person arm.

Feedback indicated that peer interaction, particularly interacting with peer coaches and learning about others experiences, seemed to be a popular component of interventions among patients. Conversely, the main barriers to use were physical limitations and pain and, when attending in-person sessions, time constraints and travel burden (Aschbrenner et al., 2015; Muralidharan et al., 2018; Olmos-Ochoa et al., 2019). Some participants attending in-person sessions also found it difficult to engage with new people (Olmos-Ochoa et al., 2019; Young et al., 2017). Other less commonly reported barriers included concerns about their environment and safety (for in-person interventions), financial barriers, control over food preparation, and lack of support from others (Olmos-Ochoa et al., 2019). Concerning wearables, participants found them helpful for setting goals, motivation, and useful for self-monitoring (Aschbrenner et al., 2015; Naslund, Aschbrenner, Barre, & Bartels, 2015a; Naslund, Aschbrenner, & Bartels, 2016). Some participants did experience frustration due to forgetting to wear pedometers (Young et al., 2017) and the cost of wearables was identified as a barrier in one study (Naslund et al., 2015a, 2015b).

*Usability.* None of the studies measured usability using formally validated scales, complicating evaluation. Some participants did comment that they found wearables easy to use (Naslund et al., 2015a, 2015b). Notably some participants did experience technical issues when using equipment (Young et al., 2017) or logging into digital interfaces for the first time. Peer coaches were noted as helpful in combatting such issues. Of note, in an intervention that involved participants playing physically active video games, 69% of participants completed the intervention using Kinect, although 85% reported would not have done so without technical support (Campos et al., 2015). Thus suggesting that without support this is not acceptable for those with an SMI and technical support would be required for real-world implementation in mental health settings (Campos et al., 2015).

*Behaviour and health outcomes.* Nine studies assessed the impact of the digital interventions on physical activity and/or weight loss (Table 5). Five studies showed at least some promising results, with two studies in particular reporting participants lost at least 5% of their body weight and clinically significant reductions in cardiovascular risk (⩾5% weight loss or improved fitness) (Aschbrenner et al., 2016a, 2021). Additionally Aschbrenner et al. (2016a, 2016b) reported 17% of participants showed clinical significant improvements in cardiovascular fitness. Two interventions lead to increases in physical activity (Macias et al., 2015; Muralidharan et al., 2018). Only one study, which used a web tool designed to help patients set goals, monitor their progress, and receive feedback via a mental health nurse (Looijmans et al., 2019) found no significant reductions in body mass index (BMI) or waist circumference at 6/12-month follow-ups.

Papers investigating a digital intervention called ‘WebMOVE’ (Muralidharan et al., 2018; Young et al., 2017) reported more weight loss than the in-person comparator intervention (MOVE-SMI). Both provided pedometers and access to peer coaches and comprised of the same educational content, differing only in delivery mode. However, in another study, both individual mHealth coaching and in-person HBC were similarly effective; with both groups achieving clinically significant weight loss and reduction in cardiovascular risk at 6 and 12 months (Aschbrenner et al., 2021).

Only one study looked at the effect of digital HBC on mental health (Campos et al., 2015) and found slight, non-significant improvements in these domains.

#### Other health behaviours

Two studies delivered HBC through web-based interventions, with one promoting the treatment for substance use disorder (SUD) (Hammond et al., 2020) and the other changing attitudes towards health behaviours as a route to behavioural changes (Melamed et al., 2022). One study targeted sleep, which used an app (Taylor et al., 2022).

*Feasibility, acceptability, effectiveness, and outcomes of other interventions.* Across the three studies, recruitment appeared to be challenging, with issues around screening and ineligibility (Table 6). Retention for the primary endpoint of theses interventions was excellent, ranging from 93% to 97%, though longer term follow-up (24 weeks) dropped to 40% in one study (Melamed et al., 2022).

Adherence to interventions varied across the three studies (58–100%). Adherence was highest for the sleep intervention. Overall participants had positive experiences, but many felt the 6-week intervention was not long enough and needed more variety of content and games (Taylor et al., 2022).

The app-based sleep intervention had a large effect on behaviour (sleep) and a small-to-medium effect on mental health (Taylor et al., 2022). The attitude-focused intervention led to positive changes in individual attitudes but did not ultimately change behaviours (Melamed et al., 2022).

The SUD intervention was rated highly across several measures, including acceptability (Hammond et al., 2020). At the end of the web intervention period, similar rates of participants had enrolled in SUD treatment, at 30 days post discharge, as that observed under treatment-as-usual conditions.

## Discussion

This paper reviewed 36 studies and systematically identified 29 digital HBC (with overlap of components for some of the physical activity interventions) for people with SMI. Feasibility, acceptability, and outcomes of interventions were evaluated and intervention components and strategies which were preferred by people with SMI were identified. Overall, 70% of the studies established support for the acceptability and/or feasibility of digital behavioural change interventions. However, themes around the need for human support for both digital literacy/navigation and engagement were common across all clinical targets. Given the pilot nature of studies and the heterogeneous outcomes, it is not possible to determine an effect size estimate, but current evidence shows that these interventions do have the potential to change health behaviours.

Across the studies reviewed, there was a relatively consistent result that digital interventions to change behaviours are both feasible and acceptable for use among people with SMI. This is an important finding, due to the large health disparity among this group and insufficient resources in mental healthcare settings to provide lifestyle interventions in mental healthcare settings (Firth et al., 2019). Despite concerns about smartphone use as a main barrier to digital interventions in this population, the majority of participants with SMI reported digital interventions were easy to use and several studies even reported participants completed additional modules or sessions voluntarily (Aschbrenner, Naslund, Shevenell, Mueser, & Bartels, 2016b; Aschbrenner et al., 2016a; Brunette et al., 2016, 2018). It is important to note some participants did struggle with accessibility, internet access, and/or needed additional support, in particular for those with limited experience using technology (Campos et al., 2015; Ferron et al., 2011; Naslund et al., 2016; Olmos-Ochoa et al., 2019; Taylor et al., 2022; Vilardaga et al., 2016, 2019; Young et al., 2017). Promisingly those with little experience using digital platforms could use them after assistance from peer coaches (Olmos-Ochoa et al., 2019; Young et al., 2017) or training sessions (Brunette et al., 2016). Therefore, to reduce the digital divide in future, it would be crucial to have human support available in mental healthcare settings to facilitate use, such as digital navigators (Sylvia et al., 2021; Wisniewski, Gorrindo, Rauseo-Ricupero, Hilty, & Torous, 2020; Wisniewski & Torous, 2020).

It is important to mention participants recruited from veteran centres and inpatient settings had high rates of ineligible participants, which may limit the generalisability of the results to other patient populations and settings. Further, some of the digital interventions (such as Microsoft, iCOMMIT, and LTQ) are not publicly readily available to use. Two interventions financially compensated participants for ongoing engagement, which may not be sustainable in real-world healthcare services (Linardon & Fuller-Tyszkiewicz, 2020).

Compared to in-person interventions, digital HBC had benefits such as greater adherence, lower resource intensity, and the potential for non-clinical staff to deliver them (Aschbrenner et al., 2021). Further, the outcomes/changes in behaviour from digital interventions seemed similar to in-person interventions of the same content (Muralidharan et al., 2018; Olmos-Ochoa et al., 2019; Young et al., 2017). Such findings are promising given the lack of capacity in mental healthcare services for in-person HBC (Ayerbe et al., 2018; Bailey et al., 2019). However, future work is required to compare the effectiveness of delivering an intervention digitally *v.* non-digitally to people with SMI.

Therefore, digital HBC are poised to play a crucial role in the near future. Digital interventions can also increase engagement and overcome socioeconomic and barrier issues reported by participants regarding the in-person elements of the multi-component interventions.

Peer/social support – offline and online – was perceived positively among many of the physical activity interventions (Aschbrenner et al., 2015; Macias et al., 2015; Muralidharan et al., 2018; Young et al., 2017) and, from the interviews, social support was a strongly desired element for smoking cessation apps (Gowarty, Aschbrenner, & Brunette, 2021a; Gowarty et al., 2021b; Klein et al., 2019). Also, design features and content that made platforms more interactive, usable, and tailored to those with SMI enhanced engagement (Aschbrenner et al., 2016b; Browne et al., 2021; Brunette et al., 2016, 2018, 2020; Klein et al., 2019; Naslund et al., 2015a, 2015b; Taylor et al., 2022; Vilardaga et al., 2020).

With regards to behavioural change techniques, it appears that setting goals and reviewing progress may not be enough to change behaviour for people with SMI. Setting diet and physical activity goals, behavioural monitoring, and receiving feedback from health professionals failed to reduce BMI or waist circumference at 6/12-month follow-ups in one study (Looijmans et al., 2019). In contrast, interventions that involved exercise sessions, information about preparing healthy meals, wearables, provided rewards/trophies or had social support, led to weight loss for the majority of participants (Aschbrenner et al., 2016a, 2016b, 2021; Muralidharan et al., 2018; Naslund, Aschbrenner, Marsch, McHugo, & Bartels, 2018; Young et al., 2017). Previous research has shown that demonstrating exercises at home yielded large impacts on physical activity in low-income groups (Bull et al., 2018). Further research would be required to determine the feasibility and acceptability of digital home workouts in people with SMI.

People with SMI appear more amenable to HBC tailored to consider their needs. This review highlighted examples where digital HBC developed for those with SMI were found to have superior outcomes, including higher rates of smoking abstinence and/or greater reduction in cigarettes smoked (Browne et al., 2021; Brunette et al., 2018), fewer relapses (Vilardaga et al., 2020), and enhanced usability (Brunette et al., 2020). For example, with smoking interventions, tailoring could mean normalising relapses and integrating their mental health symptomology, while with physical activity interventions considering the physical limitations of people with SMI could be important (Aschbrenner et al., 2015; Klein et al., 2019; Muralidharan et al., 2018; Olmos-Ochoa et al., 2019).

## Strengths and limitations

A strength of this study was the comprehensive nature of the methods, which applied a systematic approach and broad search terms to capturing digital HBC for people with SMI, including various study designs. Although only one reviewer was responsible for an initial screening at title and abstract stage, this was done only to remove the obviously ineligible articles swiftly (i.e. those in which no part of the title or abstract indicated relevance to this review). Any study with an indication of eligibility from title/abstract content was subject to full-text screening, conducted by two reviewers. Given the auxiliary search methods conducted alongside the main search, we are confident this review captures the relevant published literature on this nascent but growing topic. However, a key limitation is that due to the preliminary nature of most studies conducted so far (which were largely focused on feasibility, or pilot studies with small-sample sizes consisting of mostly, if not all, Caucasian participants), the research may be too nascent at present to draw any definitive conclusions on the effectiveness of digital approaches for health promotion in SMI. Additionally due to the short-term follow-up of most studies (<4 months), the degree of engagement with digital interventions over longer durations is unknown.

While this review was able to summarise the acceptability/feasibility of digital HBC from a range of different metrics in SMI samples, a further limitation is that many of the included studies were conducted in the United States, which may limit generalisability to other healthcare systems. Furthermore, most studies only recruited participants who had the ability and/or interest in using digital technologies, making it hard to determine the actual feasibility of such approaches, across the entire clinical populations of those treated for SMI (i.e. beyond those individuals who are eligible and willing to join the reviewed studies, to begin with).

## Conclusions and future research

Current results suggest digital HBC overall are acceptable and useful for people with SMI, but some individuals may need extra support with technology. The effectiveness of these interventions has yet to be fully established. Nonetheless, there are many provisional findings of digital technologies can result in positive HBC among people with SMI, and even better engagement when compared with some in-person intervention components. To ensure accessibility and usability, the design process of digital interventions should aim to involve people with SMI throughout. Future research should also examine the cost-effectiveness and implementation of digital HBC for promoting health behaviours into real-world clinical settings and healthcare systems for people with SMI.

## References

[ref1] Arigo, D., Jake-Schoffman, D. E., Wolin, K., Beckjord, E., Hekler, E. B., & Pagoto, S. L. (2019). The history and future of digital health in the field of behavioral medicine. Journal of Behavioral Medicine, 42, 67–83. 30825090 10.1007/s10865-018-9966-z PMC6644720

[ref2] Aschbrenner, K. A., Naslund, J. A., Barre, L. K., Mueser, K. T., Kinney, A., & Bartels, S. J. (2015). Peer health coaching for overweight and obese individuals with serious mental illness: Intervention development and initial feasibility study. Translational Behavioral Medicine, 5(3), 277–284. 26327933 10.1007/s13142-015-0313-4 PMC4537456

[ref3] Aschbrenner, K. A., Naslund, J. A., Gorin, A. A., Mueser, K. T., Browne, J., Wolfe, R. S., … Bartels, S. J. (2021). Group lifestyle intervention with mobile health for young adults with serious mental illness: A randomized controlled trial. Psychiatric Services, 73(2), 141–148. 34189933 10.1176/appi.ps.202100047 PMC11453118

[ref4] Aschbrenner, K. A., Naslund, J. A., Shevenell, M., Kinney, E., & Bartels, S. J. (2016a). A pilot study of a peer-group lifestyle intervention enhanced with mHealth technology and social media for adults with serious mental illness. The Journal of Nervous and Mental Disease, 204(6), 483. 27233056 10.1097/NMD.0000000000000530 PMC4887192

[ref5] Aschbrenner, K. A., Naslund, J. A., Shevenell, M., Mueser, K. T., & Bartels, S. J. (2016b). Feasibility of behavioral weight loss treatment enhanced with peer support and mobile health technology for individuals with serious mental illness. Psychiatric Quarterly, 87(3), 401–415. 26462674 10.1007/s11126-015-9395-x PMC4929042

[ref6] Ayerbe, L., Forgnone, I., Foguet-Boreu, Q., González, E., Addo, J., & Ayis, S. (2018). Disparities in the management of cardiovascular risk factors in patients with psychiatric disorders: A systematic review and meta-analysis. Psychological Medicine, 48(16), 2693–2701. 29490716 10.1017/S0033291718000302

[ref7] Bailey, J. M., Bartlem, K. M., Wiggers, J. H., Wye, P. M., Stockings, E. A., Hodder, R. K., … Dray, J. A. (2019). Systematic review and meta-analysis of the provision of preventive care for modifiable chronic disease risk behaviours by mental health services. Preventive Medicine Reports, 16, 100969. 31497500 10.1016/j.pmedr.2019.100969 PMC6718945

[ref8] Balaskas, A., Doherty, G., Schueller, S. M., & Cox, A. L. (2021). Ecological momentary interventions for mental health: A scoping review. PLoS ONE, 16(3 March), e0248152. doi: 10.1371/journal.pone.0248152 33705457 PMC7951936

[ref9] Bennett, G. G., & Glasgow, R. E. (2009). The delivery of public health interventions via the internet: Actualizing their potential. Annual Review of Public Health, 30(1), 273–292. 10.1146/annurev.publhealth.031308.100235 19296777

[ref10] Borzekowski, D. L., Leith, J., Medoff, D. R., Potts, W., Dixon, L. B., Balis, T., … Himelhoch, S. (2009). Use of the internet and other media for health information among clinic outpatients with serious mental illness. Psychiatric Services, 60(9), 1265–1268. 19723745 10.1176/ps.2009.60.9.1265

[ref11] Browne, J., Halverson, T. F., & Vilardaga, R. (2021). Engagement with a digital therapeutic for smoking cessation designed for persons with psychiatric illness fully mediates smoking outcomes in a pilot randomized controlled trial. Translational Behavioral Medicine, 11(9), 1717–1725. doi: 10.1093/tbm/ibab100 34347865 PMC8571710

[ref12] Brunette, M. F., Ferron, J. C., Devitt, T., Geiger, P., Martin, W. M., Pratt, S…McHugo, G. J. (2012). Do smoking cessation websites meet the needs of smokers with severe mental illnesses? Health Education Research, 27(2), 183–190. 21987478 10.1093/her/cyr092 PMC6281343

[ref13] Brunette, M. F., Ferron, J. C., Geiger, P., Guarino, S., Pratt, S. I., Lord, S. E., …Adachi-Mejia, A. (2019). Pilot study of a mobile smoking cessation intervention for low-income smokers with serious mental illness. Journal of Smoking Cessation, 14(4), 203–210. 10.1017/jsc.2019.7 PMC11910196 40092714

[ref14] Brunette, M. F., Ferron, J. C., Gottlieb, J., Devitt, T., & Rotondi, A. (2016). Development and usability testing of a web-based smoking cessation treatment for smokers with schizophrenia. Internet Interventions, 4, 113–119. doi: 10.1016/j.invent.2016.05.003 30135797 PMC6096117

[ref15] Brunette, M. F., Ferron, J. C., McGurk, S. R., Williams, J. M., Harrington, A., Devitt, T., & Xie, H. (2020). Brief, web-based interventions to motivate smokers with schizophrenia: Randomized controlled trial. JMIR Mental Health, 7(2), e16524. doi: 10.2196/16524 32039811 PMC7055792

[ref16] Brunette, M. F., Ferron, J. C., McHugo, G. J., Davis, K. E., Devitt, T. S., Wilkness, S. M., & Drake, R. E. (2011). An electronic decision support system to motivate people with severe mental illnesses to quit smoking. Psychiatric Services, 62(4), 360–366. 21459986 10.1176/ps.62.4.pss6204_0360

[ref17] Brunette, M. F., Ferron, J. C., Robinson, D., Coletti, D., Geiger, P., Devitt, T., … Greene, M. A. (2018). Brief web-based interventions for young adult smokers with severe mental illnesses: A randomized, controlled pilot study. Nicotine and Tobacco Research, 20(10), 1206–1214. 29059417 10.1093/ntr/ntx190 PMC6121912

[ref18] Bull, E. R., McCleary, N., Li, X., Dombrowski, S. U., Dusseldorp, E., & Johnston, M. (2018). Interventions to promote healthy eating, physical activity and smoking in low-income groups: A systematic review with meta-analysis of behavior change techniques and delivery/context. International Journal of Behavioral Medicine, 25(6), 605–616. 30003476 10.1007/s12529-018-9734-z PMC6244564

[ref19] Campos, C., Mesquita, F., Marques, A., Trigueiro, M. J., Orvalho, V., & Rocha, N. B. (2015). Feasibility and acceptability of an exergame intervention for schizophrenia. Psychology of Sport and Exercise, 19, 50–58.

[ref20] Carney, R., Cotter, J., Bradshaw, T., Firth, J., & Yung, A. R. (2016). Cardiometabolic risk factors in young people at ultra-high risk for psychosis: A systematic review and meta-analysis. Schizophrenia Research, 170(2–3), 290–300. 26794596 10.1016/j.schres.2016.01.010

[ref21] Ferron, J. C., Brunette, M. F., McHugo, G. J., Devitt, T. S., Martin, W. M., & Drake, R. E. (2011). Developing a quit smoking website that is usable by people with severe mental illnesses. Psychiatric Rehabilitation Journal, 35(2), 111. doi: 10.2975/35.2.2011.111.116 22020840

[ref22] Firth, J., Cotter, J., Torous, J., Bucci, S., Firth, J. A., & Yung, A. R. (2016). Mobile phone ownership and endorsement of ‘mHealth’ among people with psychosis: A meta-analysis of cross-sectional studies. Schizophrenia Bulletin, 42(2), 448–455. 26400871 10.1093/schbul/sbv132 PMC4753601

[ref23] Firth, J., Siddiqi, N., Koyanagi, A., Siskind, D., Rosenbaum, S., Galletly, C., … Carvalho, A. F. (2019). The Lancet Psychiatry Commission: A blueprint for protecting physical health in people with mental illness. The Lancet Psychiatry, 6(8), 675–712. 31324560 10.1016/S2215-0366(19)30132-4

[ref24] Firth, J., Solmi, M., Wootton, R. E., Vancampfort, D., Schuch, F. B., Hoare, E., … Jackson, S. E. (2020). A meta-review of ‘lifestyle psychiatry’: The role of exercise, smoking, diet and sleep in the prevention and treatment of mental disorders. World Psychiatry, 19(3), 360–380. 32931092 10.1002/wps.20773 PMC7491615

[ref25] Gowarty, M. A., Aschbrenner, K. A., & Brunette, M. F. (2021a). Acceptability and usability of mobile apps for smoking cessation among young adults with psychotic disorders and other serious mental illness. Frontiers in Psychiatry, 12, 656538. doi: 10.3389/fpsyt.2021.656538 34025477 PMC8138181

[ref26] Gowarty, M. A., Longacre, M. R., Vilardaga, R., Kung, N. J., Gaughan-Maher, A. E., & Brunette, M. F. (2021b). Usability and acceptability of two smartphone apps for smoking cessation among young adults with serious mental illness: Mixed methods study. JMIR Mental Health, 8(7), e26873. doi: 10.2196/26873 34255699 PMC8295834

[ref27] Greenhalgh, T., Wherton, J., Papoutsi, C., Lynch, J., Hughes, G., Hinder, S., … Shaw, S. (2017). Beyond adoption: A new framework for theorizing and evaluating nonadoption, abandonment, and challenges to the scale-up, spread, and sustainability of health and care technologies. Journal of Medical Internet Research, 19(11), e8775. 10.2196/jmir.8775 PMC5688245 29092808

[ref28] Hammond, A. S., Antoine, D. G., Stitzer, M. L., & Strain, E. C. (2020). A randomized and controlled acceptability trial of an internet-based therapy among inpatients with co-occurring substance use and other psychiatric disorders. Journal of Dual Diagnosis, 16(4), 447–454. 32701419 10.1080/15504263.2020.1794094

[ref29] Heffner, J. L., Kelly, M. M., Waxmonsky, J., Mattocks, K., Serfozo, E., Bricker, J. B., … Ostacher, M. (2020). Pilot randomized controlled trial of web-delivered acceptance and commitment therapy versus smokefree. gov for smokers with bipolar disorder. Nicotine and Tobacco Research, 22(9), 1543–1552. 31883336 10.1093/ntr/ntz242 PMC7443589

[ref30] Heffner, J. L., Mull, K. E., Watson, N. L., Mcclure, J. B., & Bricker, J. B. (2018). Smokers with bipolar disorder, other affective disorders, and no mental health conditions: comparison of baseline characteristics and success at quitting in a large 12-month behavioral intervention randomized trial. Drug and Alcohol Dependence, 193, 35–41. 30340143 10.1016/j.drugalcdep.2018.08.034 PMC6239897

[ref31] Hyzy, M., Bond, R., Mulvenna, M., Bai, L., Dix, A., Leigh, S., & Hunt, S. (2022). System usability scale benchmarking for digital health apps: Meta-analysis. JMIR mHealth and uHealth, 10(8), e37290. 35980732 10.2196/37290 PMC9437782

[ref32] Jacob, C., Sezgin, E., Sanchez-Vazquez, A., & Ivory, C. (2022). Sociotechnical factors affecting patients’ adoption of mobile health tools: Systematic literature review and narrative synthesis. JMIR mHealth and uHealth, 10(5), e36284. 35318189 10.2196/36284 PMC9121221

[ref33] Klein, P., Lawn, S., Tsourtos, G., & van Agteren, J. (2019). Tailoring of a smartphone smoking cessation app (kick.it) for serious mental illness populations: Qualitative study. JMIR Human Factors, 6(3), e14023. doi: 10.2196/14023 31482850 PMC6754228

[ref34] Lewis, J. R. (1994). Sample sizes for usability studies: Additional considerations. Human Factors, 36(2), 368–378. 8070799 10.1177/001872089403600215

[ref35] Liberati, A., Altman, D. G., Tetzlaff, J., Mulrow, C., Gøtzsche, P. C., Ioannidis, J. P., … Moher, D. (2009). The PRISMA statement for reporting systematic reviews and meta-analyses of studies that evaluate health care interventions: Explanation and elaboration. Journal of Clinical Epidemiology, 62(10), e1–e34. 19631507 10.1016/j.jclinepi.2009.06.006

[ref36] Moher, D., Liberati, A., Tetzlaff, J., Altman, D. G. & PRISMA Group. (2009). Preferred reporting items for systematic reviews and meta-analyses: the PRISMA statement. PLoS Medicine, 6(7), e1000097. 10.1371/journal.pmed.1000097 PMC2707599 19621072

[ref37] Linardon, J., & Fuller-Tyszkiewicz, M. (2020). Attrition and adherence in smartphone-delivered interventions for mental health problems: A systematic and meta-analytic review. Journal of Consulting and Clinical Psychology, 88(1), 1. 31697093 10.1037/ccp0000459

[ref38] Looijmans, A., Jörg, F., Bruggeman, R., Schoevers, R. A., & Corpeleijn, E. (2019). Multimodal lifestyle intervention using a web-based tool to improve cardiometabolic health in patients with serious mental illness: Results of a cluster randomized controlled trial (LION). BMC Psychiatry, 19(1), 1–12. 31690281 10.1186/s12888-019-2310-5 PMC6833253

[ref39] Macias, C., Panch, T., Hicks, Y. M., Scolnick, J. S., Weene, D. L., Öngür, D., & Cohen, B. M. (2015). Using smartphone apps to promote psychiatric and physical well-being. Psychiatric Quarterly, 86(4), 505–519. 25636496 10.1007/s11126-015-9337-7

[ref40] Mazereel, V., Detraux, J., Vancampfort, D., Van Winkel, R., & De Hert, M. (2020). Impact of psychotropic medication effects on obesity and the metabolic syndrome in people with serious mental illness. Frontiers in Endocrinology, 11, 573479. 33162935 10.3389/fendo.2020.573479 PMC7581736

[ref41] Medenblik, A. M., Mann, A. M., Beaver, T. A., Dedert, E. A., Wilson, S. M., Calhoun, P. S., & Beckham, J. C. (2020). Treatment outcomes of a multi-component mobile health smoking cessation pilot intervention for people with schizophrenia. Journal of Dual Diagnosis, 16(4), 420–428. 32735514 10.1080/15504263.2020.1797259 PMC8356481

[ref42] Melamed, O., Voineskos, A., Vojtila, L., Ashfaq, I., Veldhuizen, S., Dragonetti, R., … Selby, P. (2022). Technology-enabled collaborative care for youth with early psychosis: Results of a feasibility study to improve physical health behaviours. Early Intervention in Psychiatry, 16(10), 1143–1151. 35103380 10.1111/eip.13266

[ref43] Muralidharan, A., Niv, N., Brown, C. H., Olmos-Ochoa, T. T., Fang, L. J., Cohen, A. N., … Young, A. S. (2018). Impact of online weight management with peer coaching on physical activity levels of adults with serious mental illness. Psychiatric Services, 69(10), 1062–1068. 30041588 10.1176/appi.ps.201700391 PMC6611674

[ref44] Naslund, J. A., Aschbrenner, K. A., Barre, L. K., & Bartels, S. J. (2015a). Feasibility of popular m-Health technologies for activity tracking among individuals with serious mental illness. Telemedicine and e-Health, 21(3), 213–216. 25536190 10.1089/tmj.2014.0105 PMC4365437

[ref45] Naslund, J. A., Aschbrenner, K. A., & Bartels, S. J. (2016). Wearable devices and smartphones for activity tracking among people with serious mental illness. Mental Health and Physical Activity, 10, 10–17. 27134654 10.1016/j.mhpa.2016.02.001 PMC4845759

[ref46] Naslund, J. A., Aschbrenner, K. A., Marsch, L. A., McHugo, G. J., & Bartels, S. J. (2018). Facebook for supporting a lifestyle intervention for people with major depressive disorder, bipolar disorder, and schizophrenia: An exploratory study. Psychiatric Quarterly, 89(1), 81–94. 28470468 10.1007/s11126-017-9512-0 PMC5758428

[ref47] Naslund, J. A., Marsch, L. A., McHugo, G. J., & Bartels, S. J. (2015b). Emerging mHealth and eHealth interventions for serious mental illness: A review of the literature. Journal of Mental Health, 24(5), 321–332. 26017625 10.3109/09638237.2015.1019054 PMC4924808

[ref48] Olmos-Ochoa, T. T., Niv, N., Hellemann, G., Cohen, A. N., Oberman, R., Goldberg, R., & Young, A. S. (2019). Barriers to participation in web-based and in-person weight management interventions for serious mental illness. Psychiatric Rehabilitation Journal, 42(3), 220. 31081651 10.1037/prj0000363 PMC6715516

[ref49] Pape, L. M., Adriaanse, M. C., Kol, J., van Straten, A., & van Meijel, B. (2022). Patient-reported outcomes of lifestyle interventions in patients with severe mental illness: A systematic review and meta-analysis. BMC Psychiatry, 22(1), 1–27. 35418082 10.1186/s12888-022-03854-x PMC9006587

[ref50] Prochaska, J. J., Das, S., & Young-Wolff, K. C. (2017). Smoking, mental illness, and public health. Annual Review of Public Health, 38, 165. 10.1146/annurev-publhealth-031816-044618 PMC5788573 27992725

[ref51] Sylvia, L. G., Faulkner, M., Rakhilin, M., Amado, S., Gold, A. K., Albury, E. A., … Nierenberg, A. A. (2021). An online intervention for increasing physical activity in individuals with mood disorders at risk for cardiovascular disease: Design considerations. Journal of Affective Disorders, 291, 102–109. doi: 10.1016/j.jad.2021.04.094 34029880

[ref52] Taylor, K. M., Bradley, J., & Cella, M. (2022). A novel smartphone-based intervention targeting sleep difficulties in individuals experiencing psychosis: A feasibility and acceptability evaluation. Psychology and Psychotherapy: Theory, Research and Practice, 95(3), 717–737. 10.1111/papt.12395 PMC9541554 35481697

[ref53] Teasdale, S. B., Ward, P. B., Samaras, K., Firth, J., Stubbs, B., Tripodi, E., & Burrows, T. L. (2019). Dietary intake of people with severe mental illness: Systematic review and meta-analysis. The British Journal of Psychiatry, 214(5), 251–259. 30784395 10.1192/bjp.2019.20

[ref54] Thomas, J., & Harden, A. (2008). Methods for the thematic synthesis of qualitative research in systematic reviews. BMC Medical Research Methodology, 8(1), 1–10. 18616818 10.1186/1471-2288-8-45 PMC2478656

[ref55] Thomas, N., Foley, F., Lindblom, K., & Lee, S. (2017). Are people with severe mental illness ready for online interventions? Access and use of the internet in Australian mental health service users. Australasian Psychiatry, 25(3), 257–261. 28139947 10.1177/1039856217689913

[ref56] Trefflich, F., Kalckreuth, S., Mergl, R., & Rummel-Kluge, C. (2015). Psychiatric patients’ internet use corresponds to the internet use of the general public. Psychiatry Research, 226(1), 136–141. 25623020 10.1016/j.psychres.2014.12.037

[ref57] Vancampfort, D., Firth, J., Schuch, F. B., Rosenbaum, S., Mugisha, J., Hallgren, M., … De Hert, M. (2017). Sedentary behavior and physical activity levels in people with schizophrenia, bipolar disorder and major depressive disorder: A global systematic review and meta-analysis. World Psychiatry, 16(3), 308–315. 28941119 10.1002/wps.20458 PMC5608847

[ref58] Vilardaga, R., Rizo, J., Kientz, J. A., McDonell, M. G., Ries, R. K., & Sobel, K. (2016). User experience evaluation of a smoking cessation app in people with serious mental illness. Nicotine & Tobacco Research, 18(5), 1032–1038. doi: 10.1093/ntr/ntv256 26581430 PMC4900234

[ref59] Vilardaga, R., Rizo, J., Palenski, P. E., Mannelli, P., Oliver, J. A., & Mcclernon, F. J. (2020). Pilot randomized controlled trial of a novel smoking cessation app designed for individuals with co-occurring tobacco use disorder and serious mental illness. Nicotine and Tobacco Research, 22(9), 1533–1542. doi: 10.1093/ntr/ntz202 31667501 PMC7443597

[ref60] Vilardaga, R., Rizo, J., Ries, R. K., Kientz, J. A., Ziedonis, D. M., Hernandez, K., & McClernon, F. J. (2019). Formative, multimethod case studies of learn to quit, an acceptance and commitment therapy smoking cessation app designed for people with serious mental illness. Translational Behavioral Medicine, 9(6), 1076–1086. doi: 10.1093/tbm/iby097 30445507 PMC7184916

[ref61] Vilardaga, R., Rizo, J., Zeng, E., Kientz, J. A., Ries, R., Otis, C., & Hernandez, K. (2018). User-centered design of learn to quit, a smoking cessation smartphone app for people with serious mental illness. JMIR Serious Games, 6(1), e8881. doi: 10.2196/games.8881 PMC5790963 29339346

[ref62] Wilson, S. M., Thompson, A. C., Currence, E. D., Thomas, S. P., Dedert, E. A., Kirby, A. C., … Beckham, J. C. (2019). Patient-informed treatment development of behavioral smoking cessation for people with schizophrenia. Behavior Therapy, 50(2), 395–409. doi: 10.1016/j.beth.2018.07.004 30824254 PMC6400295

[ref63] Wisniewski, H., Gorrindo, T., Rauseo-Ricupero, N., Hilty, D., & Torous, J. (2020). The role of digital navigators in promoting clinical care and technology integration into practice. Digital Biomarkers, 4(1), 119–135. 33442585 10.1159/000510144 PMC7768140

[ref64] Wisniewski, H., & Torous, J. (2020). Digital navigators to implement smartphone and digital tools in care. Acta Psychiatrica Scandinavica, 141(4), 350–355. 31930477 10.1111/acps.13149 PMC7928068

[ref65] Young, A. S., Cohen, A. N., Goldberg, R., Hellemann, G., Kreyenbuhl, J., Niv, N., … Whelan, F. (2017). Improving weight in people with serious mental illness: The effectiveness of computerized services with peer coaches. Journal of General Internal Medicine, 32(1), 48–55. 28271427 10.1007/s11606-016-3963-0 PMC5359157

